# LINE-1 retrotransposons drive human neuronal transcriptome complexity and functional diversification

**DOI:** 10.1126/sciadv.adh9543

**Published:** 2023-11-01

**Authors:** Raquel Garza, Diahann A. M. Atacho, Anita Adami, Patricia Gerdes, Meghna Vinod, PingHsun Hsieh, Ofelia Karlsson, Vivien Horvath, Pia A. Johansson, Ninoslav Pandiloski, Jon Matas-Fuentes, Annelies Quaegebeur, Antonina Kouli, Yogita Sharma, Marie E. Jönsson, Emanuela Monni, Elisabet Englund, Evan E. Eichler, Molly Gale Hammell, Roger A. Barker, Zaal Kokaia, Christopher H. Douse, Johan Jakobsson

**Affiliations:** ^1^ Laboratory of Molecular Neurogenetics, Department of Experimental Medical Science, Wallenberg Neuroscience Center and Lund Stem Cell Center, BMC A11, Lund University, 221 84 Lund, Sweden.; ^2^ Aligning Science Across Parkinson’s (ASAP) Collaborative Research Network, Chevy Chase, MD, 20815, USA.; ^3^ Department of Genome Sciences, University of Washington School of Medicine, Seattle, WA, 98195, USA.; ^4^ Department of Genetics, Cell Biology, and Development, University of Minnesota Medical School, Minneapolis, MN, 55455, USA.; ^5^ Epigenetics and Chromatin Dynamics, Department of Experimental Medical Science, Wallenberg Neuroscience Center and Lund Stem Cell Center, BMC B11, Lund University, 221 84 Lund, Sweden.; ^6^ Department of Clinical Neurosciences, University of Cambridge and Department of Pathology, Cambridge University Hospitals NHS Foundation Trust, Cambridge, UK.; ^7^ Department of Clinical Neuroscience and Wellcome-MRC Cambridge Stem Cell Institute, University of Cambridge, John van Geest Centre for Brain Repair, Cambridge CB2 0PY, UK.; ^8^ Laboratory of Stem Cells and Restorative Neurology, Lund Stem Cell Center, Lund University, SE-22184 Lund, Sweden.; ^9^ Department of Clinical Sciences Lund, Division of Pathology, Lund University, Lund, Sweden.; ^10^ Howard Hughes Medical Institute, University of Washington, Seattle, WA 98195, USA.; ^11^ Institute for Systems Genetics, Department of Neuroscience and Physiology, NYU Langone Health, New York, NY 10016, USA.; ^12^ Neuroscience Institute, NYU Grossman School of Medicine, New York, NY 10016, USA.

## Abstract

The genetic mechanisms underlying the expansion in size and complexity of the human brain remain poorly understood. Long interspersed nuclear element–1 (L1) retrotransposons are a source of divergent genetic information in hominoid genomes, but their importance in physiological functions and their contribution to human brain evolution are largely unknown. Using multiomics profiling, we here demonstrate that L1 promoters are dynamically active in the developing and the adult human brain. L1s generate hundreds of developmentally regulated and cell type–specific transcripts, many that are co-opted as chimeric transcripts or regulatory RNAs. One L1-derived long noncoding RNA, *LINC01876*, is a human-specific transcript expressed exclusively during brain development. CRISPR interference silencing of *LINC01876* results in reduced size of cerebral organoids and premature differentiation of neural progenitors, implicating L1s in human-specific developmental processes. In summary, our results demonstrate that L1-derived transcripts provide a previously undescribed layer of primate- and human-specific transcriptome complexity that contributes to the functional diversification of the human brain.

## Teaser

Multi-omic assesment of L1 expression reveals a wide effect in the human brain transcriptome which carries functional potential.

## INTRODUCTION

During evolution, primate brains have expanded in size and complexity resulting in a unique level of cognitive functions. The genetic alterations responsible for this enhancement remain poorly understood (*1*–*4*). Our closest living relative, the chimpanzee, shares more than 98% of protein-coding sequences with humans, making it unlikely that species-specific protein-coding variants are the sole evolutionary drivers of brain complexity (*5*, *6*). Rather, a substantial fraction of the genetic basis for the differences in nonhuman primate and human brains likely resides in the noncoding part of the genome.

Transposable elements (TEs) make up at least 50% of the human genome (*7*). Since TEs have populated the genome through mobilization, this has resulted in major interspecies and interindividual differences in their genomic composition. Hundreds of thousands of TEs are primate specific, and several thousand of them are human specific (*8*, *9*). TEs pose a threat to genomic integrity—as their activation may result in retrotransposition events that cause deleterious mutations (*10*, *11*)—and the host has therefore evolved numerous mechanisms to prevent mobilization (*12*, *13*). In somatic human tissues such as the brain, it is thought that the vast majority of TEs is transcriptionally repressed, which correlates with the presence of DNA CpG methylation (*14*, *15*). However, TEs have the potential to be exapted, providing a benefit for the host as a source of gene regulatory elements and co-opted RNAs and peptides (*16*). For example, TEs are largely responsible for the emergence of species-specific long noncoding RNAs (lncRNAs) (*17*), which are untranslated transcripts of more than 200 nucleotides that have been implicated to control a wide variety of cellular processes (*18*).

The most abundant and only autonomously mobilizing TE family in humans is long interspersed nuclear element–1 (L1) (*19*). The human genome holds around half a million individual L1 copies, occupying ~17% of genomic DNA, including ancient fragments and evolutionarily younger full-length copies (*7*, *20*). Since L1s have colonized the human genome via a copy-and-paste mechanism in different waves, it is possible to approximate the evolutionary age of each individual L1 copy and assign them to chronologically ordered subfamilies (*21*). Only L1s with an intact 5′ untranslated region (UTR) allows for element-derived expression. However, most L1s are inactivated because of 5′ truncations and the accumulation of inactivating deletions and mutations. Full-length L1s are transcribed from an internal 5′ RNA polymerase II promoter as a bicistronic mRNA encoding two proteins, ORF1p and ORF2p, which are essential for L1 mobilization (*22*–*24*). Notably, the L1 promoter is bidirectional, and in evolutionarily young L1s, the antisense transcript encodes a small peptide, ORF0, with poorly characterized function (*25*, *26*). L1-antisense transcripts can also give rise to chimeric transcripts and act as alternative promoters for protein-coding genes (*14*, *26*).

Over the past two decades, L1 activity has been implicated in the functional regulation of the human brain, primarily based on the observation of somatic L1 retrotransposition events in the neural lineage leading to genomic mosaicism (*27*–*33*). However, it has been challenging to determine the functional impact of these events. Given their abundance and repetitive nature, L1s are difficult to study using standard molecular biology techniques. For example, estimation of L1-derived RNA expression using quantitative polymerase chain reaction (PCR)–based techniques or standard short-read RNA sequencing (RNA-seq) approaches, whether bulk or single cell, often fails to separate L1 expression originating from the L1 promoter from that of bystander transcripts that are the result of readthrough transcription (*34*). Therefore, it is still debated whether and in which cell type L1 expression occurs in the developing and adult human brain and the impact of L1s on the physiology of the human brain remains unresolved.

In this study, we have used a combination of bulk short-read, long-read, and single-nucleus RNA-seq (snRNA-seq) coupled with cleavage under targets and release using nuclease (CUT&RUN) epigenomic profiling, together with tailored bioinformatics approaches (*35*, *36*) to demonstrate that L1-derived transcripts are highly expressed in the developing and the adult human brain. We found that the bidirectional L1 promoter is dynamically active, resulting in the generation of hundreds of L1-derived transcripts that display developmental regulation and cell type specificity. We provide evidence for the expression of full-length L1s and L1s that are co-opted as regulatory RNAs or alternative promoters. One human-specific L1-derived lncRNA (L1-lncRNA), *LINC01876*, is exclusively expressed during human brain development. CRISPR interference (CRISPRi)–based silencing of *LINC01876* results in reduced size of cerebral organoids and premature differentiation of neural progenitor cells (NPCs) and neurons, suggesting that it has an important role in brain development. Together, these results demonstrate that L1-derived transcripts are abundant in the human brain where they provide an additional layer of primate- and human-specific transcriptome complexity that may have contributed to the evolution of the human brain.

## RESULTS

### L1-derived transcripts are abundant in the adult human brain

**Fig. 1.**
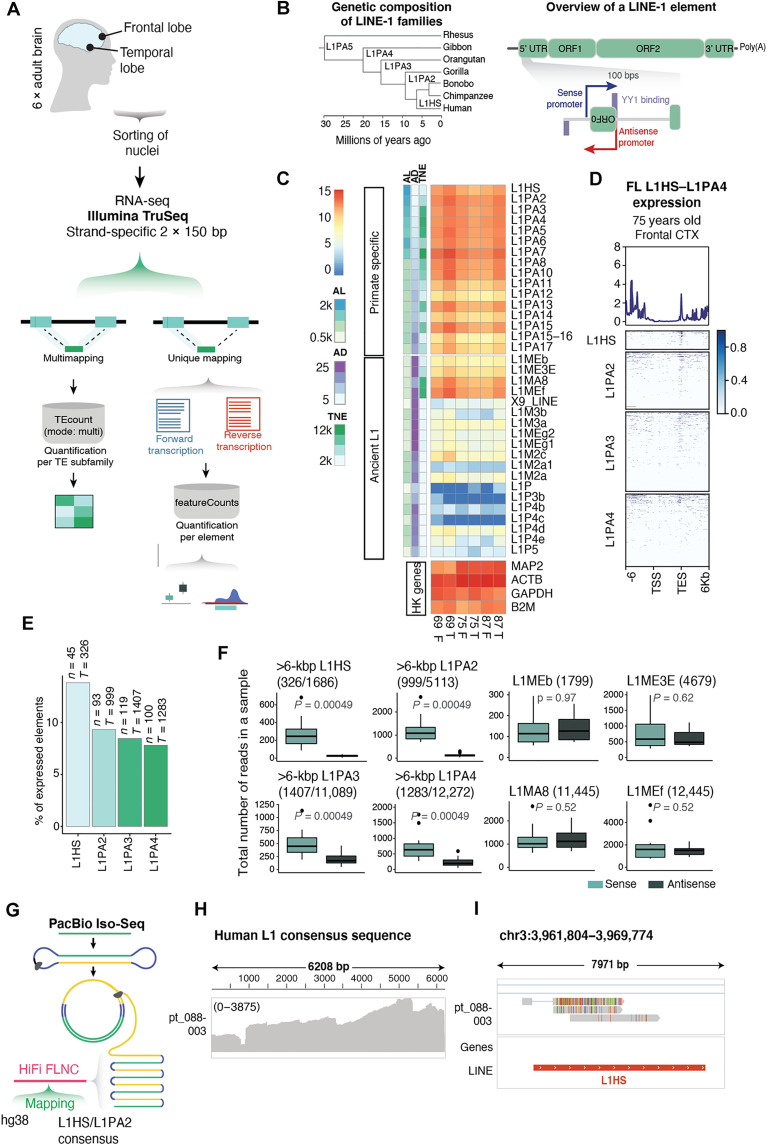
L1-derived transcripts are abundant in the adult human brain. (**A**) Schematic illustrating sample collection, sequencing strategy, and bioinformatics approach. (**B**) Left: Phylogenetic tree showing the evolutionary age of young L1 subfamilies. Right: Structure of a L1 element with a zoom-in to its 5′UTR. Arrows indicate promoters in sense (blue) and antisense (red). YY1 binding sites indicated in purple boxes (sense on top and antisense on bottom). (**C**) Expression of primate-specific L1 subfamilies compared to ancient L1 subfamilies and selected housekeeping (HK) genes as reference. Row annotation showing average length (AL), average percentage of divergence from consensus (AD), and the total number of elements (TNE) (information extracted from RepeatMasker open-4.0.5). (**D**) Expression [reads per kilobase per million mapped reads (RPKM)] over full-length (>6 kbp) L1HS, L1PA2, L1PA3, and L1PA4 plus 6-kbp flanking regions. (**E**) Percentage of expressed full-length (>6 kbp) elements (mean normalized counts, >10; see Methods) among young L1 subfamilies (*n* = number of expressed elements; *T* = total number of full-length elements). (**F**) Read counts in sense (light teal) and antisense (dark teal) per sample. First four showing full-length elements in young L1 subfamilies and last four showing ancient L1 subfamilies with a comparable number of copies. (**G**) PacBio Iso-Seq schematic and mapping approach. (**H**) Coverage of PacBio Iso-Seq library mapped to L1HS and L1PA2 consensus sequence. (**I**) Genome browser tracks showing PacBio Iso-Seq reads over the promoter region of a full-length L1HS.

To investigate the expression of L1s in the adult human brain, we obtained cortical tissue biopsies (temporal and frontal lobe) from three non-neurological deaths in people aged 69, 75, and 87 years (table S1). We sorted cell nuclei from the biopsies, extracted RNA, and used an in-house 2 × 150–base pair (bp), polyadenylate [poly(A)]-enriched stranded library preparation for bulk RNA-seq using a reduced fragmentation step to optimize library insert size for L1 analysis. These reads can be mapped uniquely and assigned to individual L1 loci, except for reads originating from a few of the youngest L1s and polymorphic L1 alleles that are not in the hg38 reference genome. We obtained ~30 million reads per sample. To quantify L1 expression, we used two different bioinformatics methodologies (Fig. 1A). First, we allowed reads to map to different locations (multimapping) and used the TEtranscripts software (*35*) in multimode to best assign these reads (fig. S1A). Second, we discarded all ambiguously mapping reads and only quantified those that map uniquely to a single location (unique mapping).

We found that L1s expressed in the adult human brain primarily belonged to primate-specific families, including both hominoid-specific (L1PA2 to L1PA4) and human-specific elements (L1HS) (Fig. 1B) (*21*). The total expression level of these subfamilies, as quantified with TEtranscripts (*35*), corresponds to expression levels of housekeeping genes (Fig. 1C). Using unique mapping, we were able to detect expression coming from hundreds of evolutionarily young L1s (Fig. 1D), including 138 full-length L1HS or L1PA2 elements (Fig. 1E). The RNA-seq signal over the full-length L1s was highly enriched at the 3′ end, which not only reflects the presence of degraded RNA in human postmortem samples and L1-mappability issues in the central part of the element but also indicates that the transcription of L1s terminates in the internal L1 polyadenylation signal (*37*). When comparing the number of reads transcribed in the same orientation as the L1s (in sense) to those in the opposite direction (in antisense), we found that most of the transcription in these regions was in sense to the L1s (Fig. 1F and fig. S1B). This suggests that most L1 transcripts originate from the L1 promoter and are not a consequence of readthrough or bystander transcription. In a few cases, we also found clear evidence of activity of the antisense L1 promoter (*26*), resulting in transcription extending out into the upstream flanking genome (fig. S1C).

To complement this analysis, we performed long-read PacBio Iso-Seq on a cortical biopsy from a deceased 84-year-old man (Fig. 1G). This allows for the identification of L1-derived transcripts that can be accurately mapped to full-length L1s and enables the identification of transcription starting sites (TSSs) and splicing events. We mapped reads [mean read length, 2.9 kilo base pairs (kbp)] to the L1HS and L1PA2 consensus sequence to which 11,120 reads mapped (of a total of 2 million reads in the library). The density of the mapped reads throughout the sequence reflected the common 5′ truncation that is present in most L1 copies in the human genome (*20*, *38*), but 1714 reads still mapped to the 5′UTR (Fig. 1H). Notably, we found several clear examples of long reads mapping to the promoter region of young full-length L1s providing further support to L1 promoter–driven expression in the adult human brain (Fig. 1I).

### L1 expression is enriched in neurons in the adult human brain

**Fig. 2.**
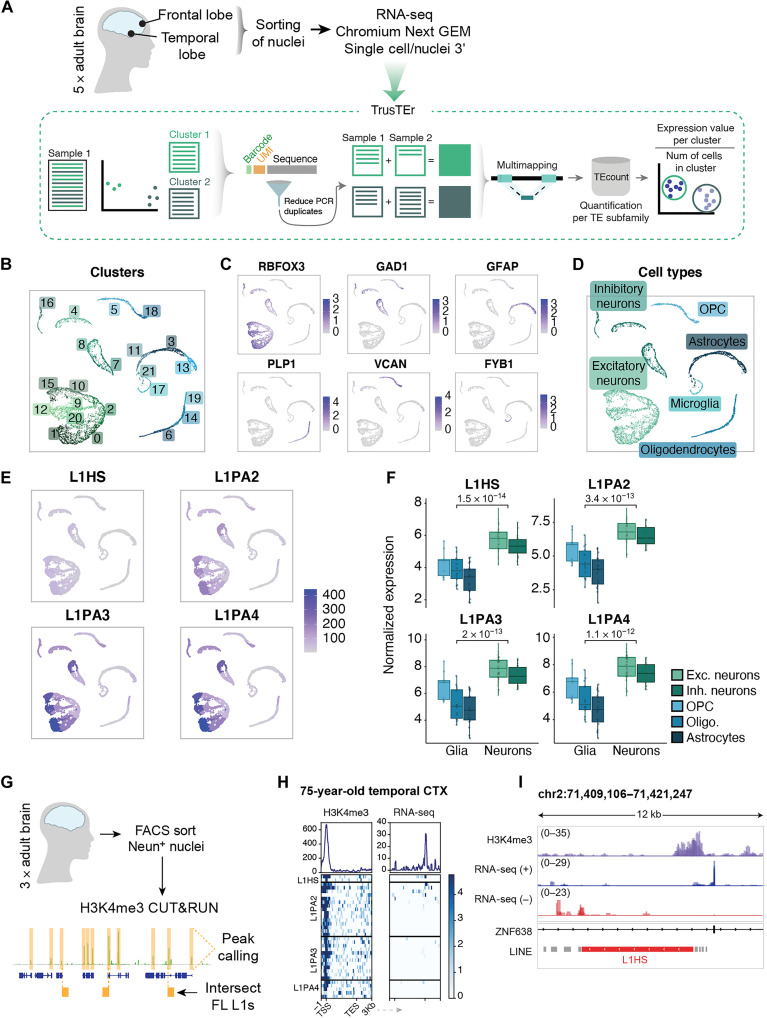
L1 expression in neurons in the adult human brain. (**A**) Schematic of sample collection, sequencing approach, and analytical bioinformatics pipeline for TE expression in single-nucleus data. (**B**) snRNA-seq: Uniform Manifold Approximation and Projection (UMAP) colored by defined clusters. (**C**) Expression of selected markers for different cell types. (**D**) UMAP colored by characterized cell types. (**E**) Pseudo-bulk cluster expression of young L1 subfamilies on UMAP. OPC, oligodendrocyte precursor cell. (**F**) Comparison of glia versus neuronal clusters per L1 family (each data point corresponds to a particular cluster expression value in a sample) (*P* value as per Wilcoxon test). (**G**) Schematic of NeuN^+^ H3K4me3 CUT&RUN in adult human brain samples and bioinformatics approach. (**H**) H3K4me3 peaks (left heatmap) over full-length L1 subfamilies (L1HS to L1PA4) and their RNA-seq signal (right heatmap). Profile plots showing sum of signal. CTX, cortex. (**I**) Genome browser tracks showing the expression of a full-length L1HS with an H3K4me3 peak on its promoter and RNA-seq signal (RPKM) split by direction of transcription (blue, forward; red, reverse).

To investigate the expression of L1s at cell type resolution, we performed snRNA-seq analysis using the 3′ 10x Chromium Platform and five of the adult cortical samples that we sequenced in bulk RNA-seq (Fig. 2A). In total, we sequenced 8089 high-quality nuclei with a mean of 3042 genes detected per cell. Unbiased clustering using Seurat resulted in 22 clusters (Fig. 2B), and on the basis of the expression of canonical gene markers, we identified excitatory neurons, inhibitory neurons, astrocytes, oligodendrocytes, oligodendrocyte precursors (OPC), and microglia at expected ratios (Fig. 2, C and D, and fig. S2A).

Quantification of L1 expression is challenging using single-cell technologies, as the number of mapped reads in a single cell falls short of accurate quantification, regardless of the mapping technique. To circumvent this limitation, we used an in-house bioinformatics pipeline allowing the analysis of L1 expression from the snRNA-seq dataset (Fig. 2A). This method uses the cell clusters determined on the basis of gene expression. Then, by back-tracing the reads from cells forming each cluster, it is possible to analyze the expression of L1s, using the TEtranscripts software (*35*) or with unique mapping, in distinct cell populations. This pseudo-bulk approach greatly increases the sensitivity of the TE analysis and enables quantitative estimation of L1 expression at single–cell type resolution (*36*).

We found clear evidence of L1 expression in the snRNA-seq data. Notably, L1 expression was higher in neurons, including both excitatory and inhibitory neurons, when compared to different glial cell types (Fig. 2, E to F, and fig. S2B). To confirm that L1s were expressed in neurons, but not in glia, we examined the transcription of each cluster per individual element using unique mapping (fig. S2B). Profile plots on reads from neurons displayed distinctive peaks over the elements, which correlated with the mappability of the L1s (fig. S2, B to D). In line with the bulk RNA-seq data, we observed that L1 expression was confined to evolutionary young elements and that the antisense signal over L1HS and L1PA2 was negligible, implying that the signal in sense of the elements is not due to readthrough or bystander transcription (fig. S2C).

To further confirm that the L1 expression in human neurons originates from the L1 promoter, we performed 5′-enriched snRNA-seq using the 10x Chromium Platform since this approach allows detection of the TSSs (fig. S2E) (*39*). We again observed the expression of evolutionary young L1s in neurons but not in glia (fig. S2, G and H), further strengthening the observation that L1 expression in human neurons originates from the L1 promoter.

### L1 promoters carry H3K4me3 in adult human neurons

The bulk and snRNA-seq analyses demonstrate that L1s are highly expressed in adult human neurons. However, because of the presence of many polymorphic L1s in the human population, it is not possible to assign this expression to individual elements with complete certainty due to the absence of these polymorphic L1s in the hg38 reference genome (*9*, *40*). To address this issue, we performed CUT&RUN epigenomic analysis (*41*) on adult human neurons to identify whether the histone mark H3K4me3, which is associated with active promoters, was present on L1s. Since the signal of this histone modification spreads to the unique flanking genomic context, this approach allows for an accurate identification of transcriptionally active individual L1 loci (*14*). To this end, we fluorescence-activated cell sorting (FACS)–isolated neuronal nuclei (NeuN^+^) from the same three human cortical biopsies used for the transcriptomic analysis and performed CUT&RUN. The resulting sequencing data were uniquely mapped, followed by peak calling and intersection with full-length L1s (Fig. 2G).

The H3K4me3 analysis identified 38 high-confidence H3K4me3 peaks located in the TSS of full-length evolutionary young L1s (Fig. 2H) (several elements were confirmed to be expressed in the bulk RNA-seq dataset). These elements represent examples of L1 transcriptional activity in adult human neurons that can be bona fide assigned to individual elements. For example, we found a full-length L1HS located in the intron of *ZNF638* as being transcriptionally active in adult human neurons (Fig. 2I).

### L1s are expressed during human brain development

**Fig. 3.**
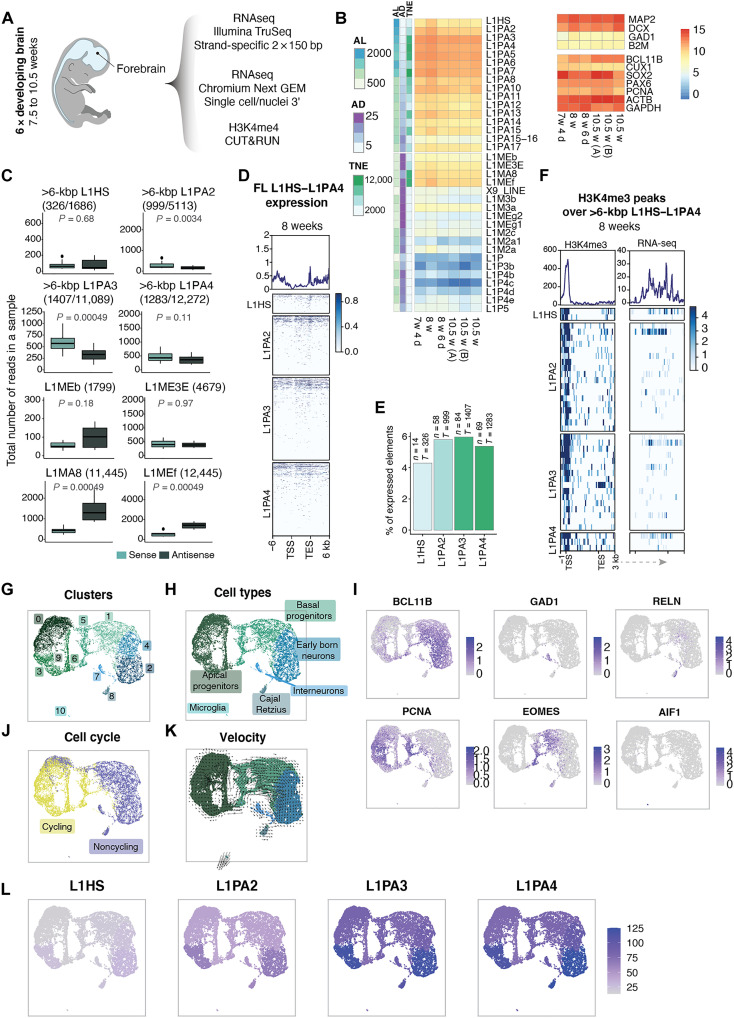
L1s are expressed in human brain development. (**A**) Schematic of sequencing strategy of fetal human forebrain samples. (**B**) Expression of primate-specific L1 subfamilies compared to ancient L1 subfamilies and selected housekeeping and development-related genes as reference. Row annotation showing average length, average percentage of divergence from consensus, and the total number of elements (information extracted from RepeatMasker open-4.0.5). (**C**) Read count in sense (light teal) and antisense (dark teal) per sample. First four boxplots showing full-length elements in young L1 subfamilies and last four showing ancient L1 subfamilies with a comparable number of copies. (**D**) Expression (RPKM) over full-length (>6 kbp) L1HS, L1PA2, L1PA3, and L1PA4 plus 6-kbp flanking regions. (**E**) Percentage of expressed full-length (>6 kbp) elements (mean normalized counts, >10; see Methods) among young L1 subfamilies (*n* = number of expressed elements; *T* = total number of full-length elements). (**F**) Detected H3K4me3 peaks (left heatmap) over full-length L1 subfamilies (L1HS to L1PA4) and RNA-seq signal (right heatmap). Profile plots showing sum of signal. (**G**) Fetal human forebrain snRNA-seq UMAP colored by cluster. (**H**) UMAP colored by cell types. (**I**) Expression of selected biomarkers for different cell types. (**J**) UMAP colored by cell cycle state (based on CellCycleScoring from Seurat). (**K**) Velocity plot colored by cell type. (**L**) Pseudo-bulk cluster expression of young L1 subfamilies on UMAP.

To investigate whether L1s are also expressed during human brain development, we analyzed six human fetal forebrain samples aged 7.5 to 10.5 weeks after conception using our multiomics approach (Fig. 3A and table S1). The bulk RNA-seq analysis demonstrated that evolutionary young L1s are expressed at levels approaching those of housekeeping genes in forebrain human development (Fig. 3B). We found no obvious difference in the magnitude of expression between the different gestational ages of the tissue. Unique mapping revealed that hundreds of different L1 loci were expressed, with the majority of these displaying sense strand enrichment, indicating an active L1 promoter (Fig. 3, C to E, and fig. S3, B and C). In line with this, the H3K4me3 analysis confirmed that several L1s carried this histone mark over the TSS, thus representing bona fide examples of unique L1 integrant expression in brain development (Fig. 3F). We also found evidence of antisense transcription initiated at the L1 TSS to the upstream genome (fig. S3D).

A notable difference when comparing the data from development to the adult brain was the expression of L1HS, which are human-specific L1s of which some retain the capacity to retrotranspose (*19*, *42*). When analyzing strand-specific expression in the developing brain samples, we found no enrichment for sense strand expression of L1HS, and we found very few L1HS expressed among the elements detected from the different subfamilies (Fig. 3, C and E). This contrasts with the adult samples where we detected clear evidence for sense-strand expression of L1HS expression and found many unique L1HS loci to be expressed (Fig. 1E). Thus, L1HS expression, which includes all elements with retrotransposition capacity (*19*, *42*), appears to be selectively silenced during human brain development.

We performed snRNA-seq on the fetal forebrain samples and sequenced 12,183 high-quality nuclei with a mean of 3818 genes detected per cell. Unbiased clustering using Seurat resulted in 11 clusters (Fig. 3G), and on the basis of the expression of canonical gene markers representing cell types present at this developmental stage, we identified apical progenitors, basal progenitors, early-born neurons, immature interneurons, Cajal Retzius cells, and microglia (Fig. 3, H and I). We also used RNA velocity (*43*) and scoring of cell cycle–related genes to further characterize this dataset (Fig. 3, J and K). These analyses revealed, in line with the existing literature, that apical progenitors represent an early proliferative neural progenitor stage that, with time, is replaced by more mature basal progenitors and postmitotic immature neurons (*44*). L1 expression levels were similar in apical progenitors, basal progenitors, and early-born neurons (Fig. 3L and fig. S3, E and F), and we found no significant correlation between L1 expression level and cell cycle state (fig. S3G). Thus, L1s are expressed in human forebrain development already at the progenitor stage, and expression is not substantially increased in differentiation and exit of the cell cycle.

### Individual L1 loci are dynamically expressed in the developing and the adult human brain

**Fig. 4.**
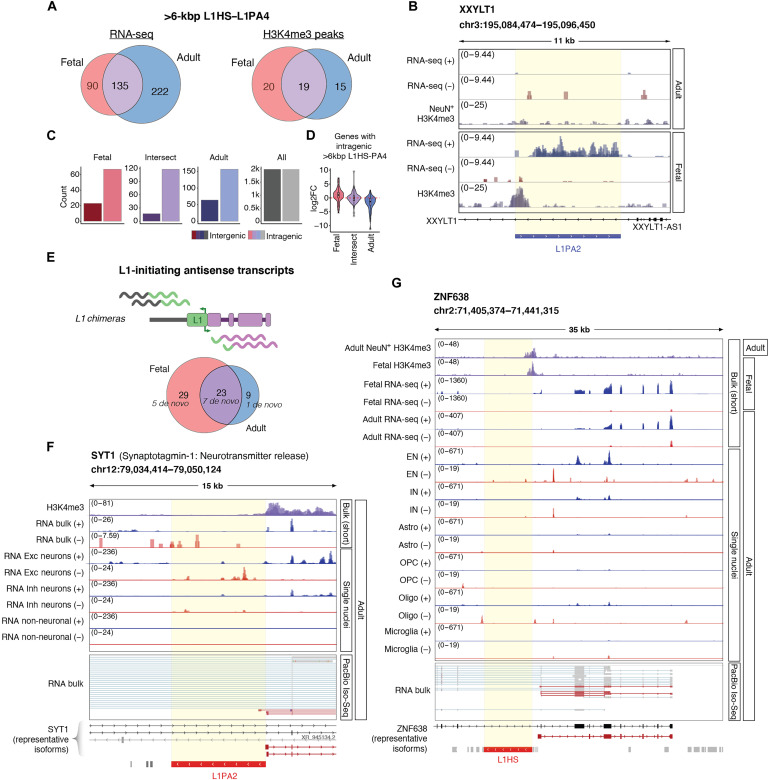
L1s are dynamically expressed in the developing and the adult human brain. (**A**) Left: Number of expressed L1HS-L1PA4 (>6 kbp) in fetal (red) and adult samples (blue) (mean normalized counts, >10; see Methods) and the number of elements found to be expressed in both datasets (intersection; purple). Right: Number of H3K4me3 peaks over L1HS-L1PA4 (>6 kbp) in fetal (red) and adult samples (blue) and the intersection between datasets (purple). (**B**) Genome browser track showing the expression of a development-specific full-length L1PA2 with an H3K4me3 peak at its promoter. (**C**) Number of intragenic (light) or intergenic (dark) L1HS to L1PA4 (>6 kbp) in fetal (red), adult (blue), or those expressed in both datasets (purple). (**D**) Log_2_FoldChange (log_2_FC) of the genes with an intragenic L1HS to L1PA4 (>6 kbp) in fetal (red), adult (blue), and the intersection (purple) [fetal versus adult (ref); DESeq2]. (**E**) L1s initiating antisense transcripts. Top: Schematic definition of L1 chimeras. Bottom: Total number of L1 chimeras expressed in fetal and adult samples. Number of the subset de novo annotated transcripts (not present in GENCODE hg38 version 38) in italics. (**F** and **G**) Genome browser tracks showing (from top to bottom): H3K4me3 CUT&RUN (samples overlayed in purple), short-read bulk RNA-seq (overlayed) split by strand (blue, forward; red, reverse), overlayed cluster expression (adult snRNA-seq) per cell type (or group of cell types), and PacBio Iso-Seq reads validating the presence of the transcript (supporting reads are highlighted in red). Annotation to the right showing data type and dataset (adult/fetal). (F) SYT1 with an antisense full-length L1PA2 at the beginning of one of its isoforms (L1 chimera). snRNA-seq tracks showing excitatory neurons (EN), inhibitory neurons (IN), and non-neuronal cell types overlayed [astrocytes, oligodendrocyte precursor cells (OPC), oligodendrocytes (Oligo), and microglia]. (G) ZNF638 with an antisense full-length L1HS as an alternative promoter (L1 chimera).

Our results demonstrate that the internal L1 promoter is active in the developing and the adult human brain resulting in the transcription of a wide panel of L1-derived transcripts. However, we noted that the developing and adult brain samples distinctly differed in the expression of individual L1 loci. When we intersected RNA-seq or H3K4me3 data from the developing and the adult brain, we found that only a minority of L1 loci were expressed in both sample types (Fig. 4A). For example, we found a full-length L1PA2 on chromosome 3 that was highly expressed in brain development but completely silent in the adult brain (Fig. 4B). Thus, the bulk of the L1 expression from unique elements was either confined to the development or the adult brain, indicating that the expression of different L1 loci depends on cellular context (*45*).

Since individual L1 loci share the same regulatory sequences, we hypothesized that the divergent expression in the developing and the adult brain is a consequence of the L1 integration site and the transcriptional activity of the nearby genome. In line with this, we noted that expressed L1 loci were highly enriched to intragenic regions (Fig. 4C). Notably, the expression of the genes in these regions clearly correlated with the expression of individual L1 loci (Fig. 4D). For example, L1s expressed uniquely during development were often located in introns of genes with a developmental specific expression pattern (Fig. 4D). Thus, this analysis indicates that the expression of individual L1 loci is governed by their integration site and the transcriptional activity of the nearby genome.

### L1-derived transcripts contribute to transcriptome complexity in human neurons

The activity of the L1 promoter in the human brain suggests that L1s are a rich potential source of primate-specific and human-specific transcripts, which, in turn, may be co-opted and contribute to transcriptome complexity and speciation. When searching our dataset, we found several such examples of co-option where L1s appear to have integrated into and modified the human transcriptome.

To investigate the presence of L1-derived transcripts, we performed de novo transcriptome assembly from the short-read bulk RNA-seq data from the fetal and adult samples. This analysis resulted in the identification of more than 60 chimeric transcripts originating from L1 promoters of which 13 represent transcripts previously not annotated in GENCODE (version 38) (table S3). When using the de novo transcript assembly in combination with our long-read RNA-seq data, we were able to validate L1 chimeras that create an alternative start site for several genes (table S3). For example, we found an L1PA2 that provides an alternative promoter for an isoform of *SYT1*. This transcript variant was supported by H3K4me3 and long-read bulk RNA-seq and was exclusively expressed in neurons as monitored by snRNA-seq (Fig. 4F). Another example was *ZNF638*, in which an L1HS serves as its alternative promoter (Fig. 4G). This isoform was supported by long-read RNA-seq, is expressed mostly in neurons, and hosts an H3K4me3 peak in both fetal and adult samples. Thus, our multiomics approach revealed several previously uncharacterized examples where L1s are integrated into the gene regulatory landscape of the developing and the mature human brain. Notably, all these L1s represent hominoid- or human-specific insertions.

**Fig. 5.**
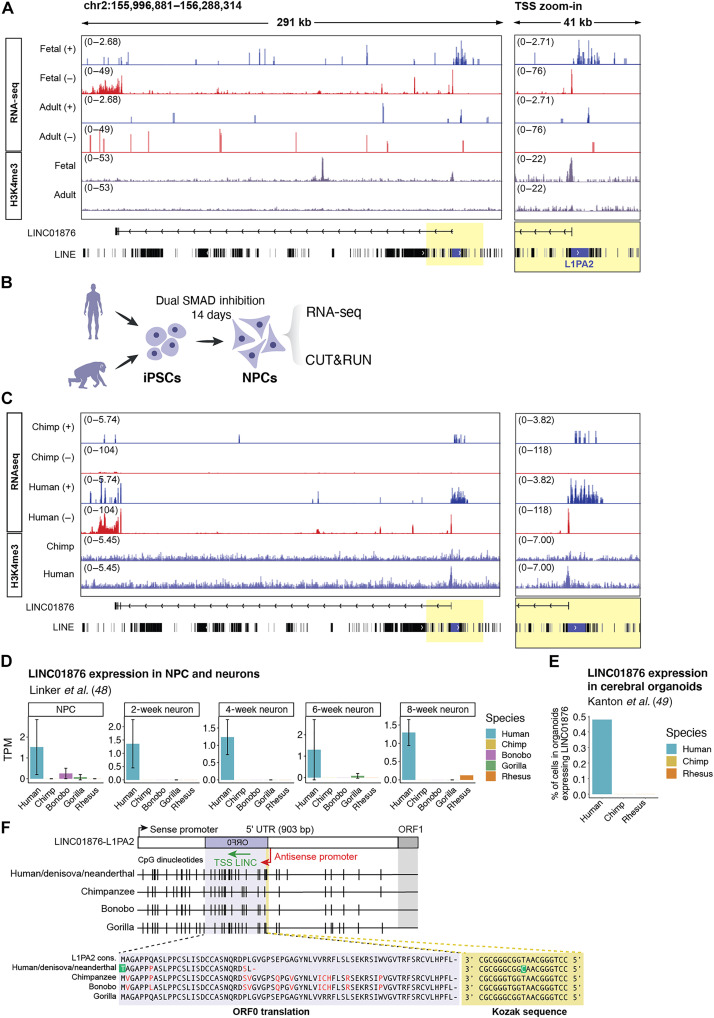
The L1-lncRNA LINC01876 is a human-specific transcript. (**A**) Genome browser tracks showing RNA-seq and H3K4me3 signal (bottom) (in purple) over L1-lncRNA in fetal and adult samples. RNA-seq signal (RPKM) split by strand (blue, forward; red, reverse). Right: A zoom-in into the TSS (highlighted in yellow). (**B**) Experimental approach for fbNPCs human and chimpanzee comparison. (**C**) Genome browser tracks showing RNA-seq and H3K4me3 signal (bottom) (in purple) over L1-lncRNA in human and chimpanzee fbNPCs. RNA-seq signal (RPKM) split by strand (blue, forward; red, reverse). Right: A zoom-in into the TSS (highlighted in yellow). (**D**) LINC01876 (L1-lncRNA) expression [transcripts per million (TPM)] from bulk RNA-seq of human, chimpanzee, bonobo, gorilla, and macaque rhesus NPCs from Linker *et al.* (*47*). (**E**) Percentage of cells expressing LINC01876 (L1-lncRNA) in human, chimpanzee, and macaque rhesus cerebral organoids from Kanton *et al.* (*48*). (**F**) Multiple sequence alignment of the L1-lncRNA L1PA2 ORF0 (highlighted in purple) in different primates and their Kozak sequence (highlighted in yellow). The TSS of the L1-lncRNA is indicated in orange.

To investigate the potential role of L1s in contributing to human brain functions, we focused on a transcriptionally active full-length L1PA2 element on chromosome 2 (6013 bp long). The L1 antisense promoter (*14*, *26*) serves as the TSS of an lncRNA: *LINC01876*. RNA-seq, snRNA-seq, and H3K4me3-CUT&RUN supported that the L1PA2 acts as an antisense promoter for this L1-lncRNA in human brain development (Fig. 5A). Notably, this expression appears to be limited to development since no *LINC01876* expression was found in the adult brain (Fig. 5A).

### L1-lncRNA LINC01876 is a human-specific transcript

L1-derived RNAs have the potential to contribute to primate and human speciation since they originate from the integration of new DNA sequences into our genome. To investigate the evolutionary conservation of the L1-lncRNA *LINC01876*, we analyzed our previously published dataset from induced pluripotent stem cells (iPSCs) derived human and chimpanzee forebrain NPCs (fbNPCs) (Fig. 5B) (*46*). We found the L1-lncRNA was highly expressed in human fbNPCs, as supported by both RNA-seq and H3K4me3 CUT&RUN data (Fig. 5C). We were not able to detect L1-lncRNA expression in chimpanzee fbNPCs. We verified the human-specific expression of this L1-lncRNA in previously published human, chimpanzee, bonobo, gorilla, and macaque RNA-seq data from NPCs and immature neurons (*47*) and snRNA-seq from human, chimpanzee, and macaque cerebral organoids (*48*) (Fig. 5, D and E). The L1-lncRNA was consistently expressed in human NPCs, immature neurons, and organoids but not in cultures obtained from other primates. Thus, the L1-lncRNA *LINC01876* appears to be a human-specific transcript that is expressed during brain development.

We performed a multiple sequence alignment of the genomic region to investigate the evolutionary time point in which the L1PA2 was inserted into the ancestral primate genome. We found that the L1PA2 insertion site is present—and identical—in human, chimpanzee, bonobo, and gorilla, but not in orangutan, macaque, or other lower species (Fig. 5F) (*49*). Thus, this L1PA2 insertion can be estimated to have occurred around 10 to 20 million years ago. To explain how the L1PA2 element drives the expression of L1-lncRNA in humans, but not in other species, we focused on its promoter region. In intact young L1s, the antisense promoter drives the expression of a small L1 peptide, ORF0 (*25*) (Fig. 1G). When comparing the antisense promoter sequences of the L1PA2 insertion between humans, chimpanzees, bonobos, and gorillas, we noticed a missense mutation (A451G) in the Kozak sequence of the ORF0 in humans (Fig. 5F). This mutation was located at the start codon resulting in a methionine to threonine (M1T) change disabling translation of the ORF0 in humans (*25*). The ORF0 was still intact in chimpanzees, bonobos, and gorillas. Denisova and Neanderthal genomes both displayed the human variant, suggesting that the nucleotide change occurred before the split of archaic human species (Fig. 5F) (*49*). This analysis indicated that it is possible that the L1-lncRNA promoter may be silenced by DNA methylation or other repressive factors in nonhuman primates due to the expression and translation of an ORF0-fusion-transcript. The L1-lncRNA *LINC01876* might escape silencing in humans as ORF0 is not translated, although the underlying mechanisms remain to be investigated.

### L1-lncRNA CRISPRi reveals an important role in neural differentiation

**Fig. 6.**
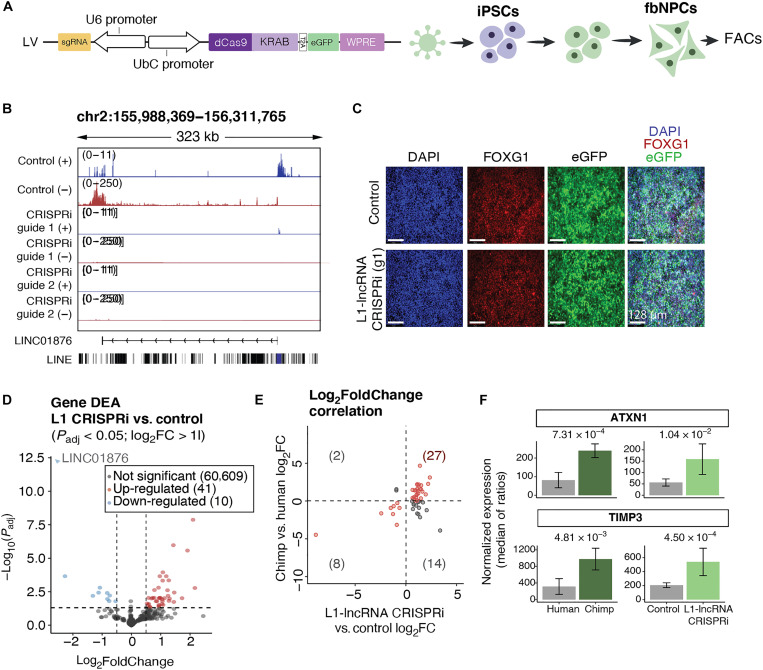
CRISPRi-silencing of the L1-lncRNA in human fbNPCs. (**A**) CRISPRi construct and schematic of the L1-lncRNA CRISPRi in fbNPCs. (**B**) Genome browser tracks showing the expression over L1-lncRNA in control (LacZ) and L1-lncRNA CRISPRi. RNA-seq signal (RPKM) split by strand (blue, forward; red, reverse). (**C**) Immunohistochemistry of forebrain (red, FOXG1) and nuclear [blue, 4′,6-diamidino-2-phenylindole (DAPI)] markers. Enhanced green fluorescent protein (eGFP) showing transfected cells (green). Scale bars, 128 μm. (**D**) Volcano plot showing differential gene expression results (DESeq2). Significantly up-regulated and down-regulated genes are highlighted in red and blue, respectively (log2FoldChange > 1, *P*_adj_ < 0.05). (**E**) Log2FoldChange of the significantly up-regulated or down-regulated genes upon L1-lncRNA CRISPRi [as highlighted in (D) in the two datasets (L1-lncRNA CRISPRi versus control and human versus chimp). Genes up-regulated or down-regulated in both datasets are highlighted in red (first and third quadrants). (**F**) Normalized expression (median of ratios; DESeq2) of two example genes up-regulated in both datasets.

To investigate the functional relevance of the L1-lncRNA *LINC01876*, we set up a CRISPRi strategy to silence *LINC01876* expression. We designed two distinct guide RNAs (gRNAs) to target unique genomic locations in the vicinity of the TSS and coexpressed these with a Krüppel-associated box (KRAB) transcriptional repressor domain fused to catalytically dead Cas9 (KRAB-dCas9) (Fig. 6A and fig. S4A). Lentiviral transduction of human iPSCs resulted in efficient, almost complete silencing of *LINC01876* upon differentiation to fbNPCs (Fig. 6B and fig. S4B), but there was no difference in differentiation capacity or expression of cell fate markers compared to controls (Fig. 6C and fig. S4C). We also found no evidence that the expression of other L1 loci was affected by the CRISPRi approach demonstrating the specificity of the silencing to the *LINC01876* locus (fig. S4, D and E). The subsequently obtained results using the two different gRNAs were indistinguishable, and thus results were pooled.

We performed RNA-seq on *LINC01876*-CRISPRi fbNPCs and analyzed the transcriptome for alterations in gene expression. We found 41 significantly up-regulated genes and 10 down-regulated genes (DESeq2; *P*_adj_ <0.05, log_2_FoldChange > 1) (Fig. 6D). As lncRNAs can act in cis or trans (*18*), we scrutinized chromosome 2 to determine whether the differentially expressed genes were located near to the lncRNA, which would indicate a cis function. We found no obvious evidence suggesting that genes in the vicinity of the L1-lncRNA on chromosome 2 were affected by the CRISPRi, indicating that the L1-lncRNA may act in trans (fig. S5F).

We noted that many of the differentially expressed genes when comparing L1-lncRNA-fbNPCs to control fbNPCs were also differentially expressed when comparing human and chimpanzee fbNPCs (*46*). Twenty-seven of the 41 up-regulated genes upon L1-lncRNA CRISPRi were more highly expressed in chimpanzee fbNPCs upon L1-lncRNA CRISPRi, and 8 of the 10 down-regulated genes after L1-lncRNA CRISPRi were expressed at lower levels in chimpanzee fbNPCs (Fig. 6E). Thus, the L1-lncRNA appeared to influence the expression of several genes that distinguish the human and chimpanzee transcriptome in neural progenitors. Notably, some of these differentially expressed genes play important roles in the human brain such as Ataxin1 (*ATXN1*), which is mutated in spinocerebellar ataxia (*50*), and tissue inhibitor of metalloproteinases 3 (*TIMP3*), which is an inhibitor of the matrix metalloproteinases that have been linked to neurodegenerative disorders (Fig. 6F) (*51*).

### L1-lncRNA LINC01876 contributes to developmental timing in cerebral organoids

**Fig. 7.**
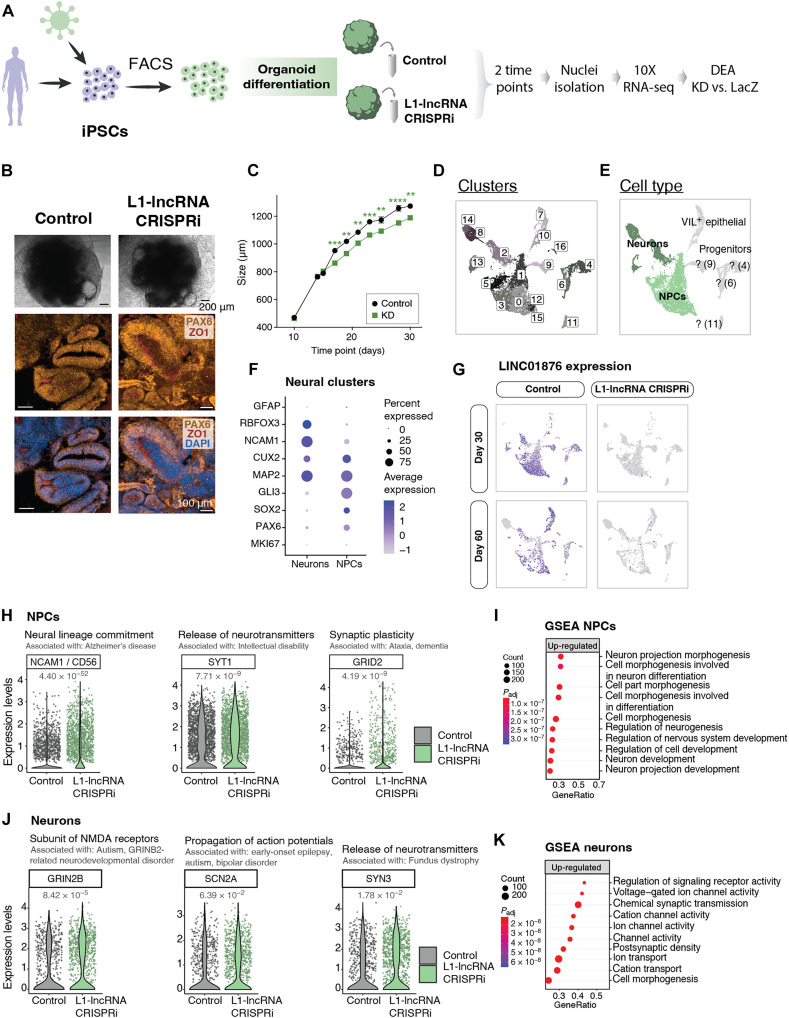
Silencing of L1-lncRNA in cerebral organoids indicates it has a role in developmental timing. (**A**) Schematic of experimental design for organoid differentiation, L1-lncRNA CRISPRi, and sequencing. DEA, Differential Expression Analysis; KD, knock down. (**B**) Bright-field pictures of iPSC-derived cerebral organoids (top). Black scale bars, 200 μm. Immunohistochemistry of PAX6 (orange), ZO1 (red), and DAPI (blue) (bottom). White scale bars, 100 μm. (**C**) Quantification of organoid diameter between days 10 and 30 (*n* = 20 to 30 organoids per time point) (mixed-effects analysis and a Sidak correction for multiple comparisons). (**D**) snRNA-seq: UMAP colored by cluster. (**E**) UMAP colored by identified cell types. Neuronal-like clusters colored in two shades of green and uncharacterized clusters or progenitor-like cells colored in grey. VIL+ , *Villin 1* positive cells. (**F**) Dot plot showing expression of neuronal and neuronal progenitor markers in the NPC and neuronal clusters. (**G**) UMAP showing expression of L1-lncRNA. (**H**) Selected examples of significantly up-regulated genes in L1-lncRNA CRISPRi NPCs (FindMarkers from Seurat; *P*_adj_ < 0.05). (**I**) Selected up-regulated terms of the gene set enrichment analysis (GSEA) over NPCs (gseGO; *P*_adj_ < 0.05). (**J**) Selected examples of significantly up-regulated genes in L1-lncRNA CRISPRi neurons (FindMarkers from Seurat; *P*_adj_ < 0.05). (**K**) Selected up-regulated terms of GSEA over neurons (gseGO; *P*_adj_ < 0.05).

To investigate the functional role of the L1-lncRNA in human brain development, we generated L1-lncRNA-CRISPRi cerebral organoids. This model allows for the study of human-specific developmental processes in three-dimensional (3D) (Fig. 7A) (*52*). We found that L1-lncRNA-CRISPRi silencing did not impair the organoid formation and the resulting organoids displayed characteristic neural rosettes after 30 days of growth, as visualized with Pax6/ZO1 staining (Fig. 7B). Quantification of organoid size throughout differentiation revealed that L1-lncRNA-CRISPRi organoids were reproducibly smaller than control organoids (Fig. 7C, table S2, and fig. S5A). This difference appeared after 2 weeks of growth and was sustained up until 1 month, which was the last time point quantified (Fig. 7C and table S2). The results were reproduced from three independent experiments using two different gRNAs (table S2).

To further evaluate the long-term molecular consequences of L1-lncRNA inhibition on human cerebral organoids, we analyzed organoids at 1 and 2 months of growth using snRNA-seq. High-quality data were generated from a total of 11,669 cells, including 6099 from L1-lncRNA-CRISPRi organoids (two gRNAs, in total 45 organoids) and 5570 from control organoids (*lacZ*-gRNA, in total 25 organoids). We performed an unbiased clustering analysis to identify and quantify the different cell types present in the organoids. Seventeen separate clusters were identified (Fig. 7D), including cerebral cells of different stages of maturation, such as NPCs and newborn neurons (Fig. 7, E and F). All the clusters contained cells from both 1 and 2 months, and we found no apparent difference in the contribution to the different clusters by L1-lncRNA-CRISPRi organoids, suggesting that the L1-lncRNA *LINC01876* does not influence developmental fate in cerebral organoids (fig. S5B).

Next, we analyzed the transcriptional difference between control and L1-lncRNA-CRISPRi organoids. We confirmed the transcriptional silencing of L1-lncRNA in all cell populations at both time points (Fig. 7G). Notably, in control (ctrl) organoids, the L1-lncRNA was expressed in NPCs but not in neurons, demonstrating that the 3D system is able to replicate an appropriate developmentally regulated expression pattern of this L1-derived transcript (Fig. 7G). We found that in the NPC population, genes linked to neuronal differentiation, such as *NCAM1*, *SYT1*, and *GRID2*, were up-regulated in L1-lncRNA-CRISPRi organoids (Fig. 7H). An unbiased gene set enrichment analysis (GSEA) of the up-regulated genes in NPCs was significantly enriched in gene ontology (GO) terms linked to neuronal differentiation (Fig. 7I). In line with this, we found that in newborn neurons, genes linked to mature neuronal functions, such as *GRIN2B*, *SCN2A*, and *SYN3*, were up-regulated in L1-lncRNA-CRISPRi organoids (Fig. 7J), and GSEA confirmed enrichment of up-regulated genes linked to neuronal maturation (Fig. 7K). These results indicate that NPCs and neurons present in organoids that lack the L1-lncRNA *LINC01876* display a more mature transcriptional profile than those found in control cerebral organoids.

Together, these results demonstrate that silencing of the L1-lncRNA *LINC01876* results in organoids that contain the same cell types as control organoids, suggesting that the L1-lncRNA does not influence developmental fate. However, we found that the L1-lncRNA organoids were smaller during early differentiation and displayed transcriptome changes in line with more mature NPCs and neurons. These observations are in line with a role for the L1-lncRNA in developmental timing since L1-lncRNA-CRISPRi organoids appear to differentiate quicker.

## DISCUSSION

L1 mobilization represents a threat to human genomic integrity, and it has therefore been assumed that L1 expression is silenced in somatic human tissues. However, the abundance and repetitive nature of L1s make their transcription difficult to precisely estimate (*34*). Previous studies have, on the basis of retrotransposition activity, indirectly indicated that L1s may be expressed in the brain (*27*–*33*). In addition, observations based on quantitative real-time PCR (qRT-PCR), Northern blots, and Cap analysis of gene expression sequencing support that full-length (defined as >6 kbp) L1 transcripts are expressed in the human brain (*53*, *54*), but these approaches lack in precision, and it has been difficult to pinpoint the expression to individual loci. Therefore, several open questions remain as follows: Which L1-loci are expressed in the human brain and in what cell types? Are L1s developmentally regulated? Do L1-derived transcripts contribute to brain functions? In this study, we resolve many of these issues through the use of a careful multiomics analysis of human tissue, combined with a customized bioinformatics pipelines. We found that L1s are highly expressed in the developing human brain and in neurons in the adult human brain.

Our data demonstrate that the expression of L1s in the developing and adult human brain is largely limited to evolutionarily young, primate specific L1s, primarily subclasses found only in hominoids. The lack of expression of more ancient L1s is likely explained by the higher burden of deletions, mutations, and genomic rearrangements of old TEs that reduce their capacity to be transcribed. A strand-specific analysis of full-length elements that contain an intact 5′ promoter revealed that the RNA-seq signal was present in sense to the L1s. We thereby confirmed that hundreds of different L1 loci are expressed and that the L1 signal is not transcriptional noise but rather that the L1 promoter drives expression. This strongly suggests that the signal is not the result of passive expression in which the L1 sequence is incorporated into another transcript (*34*). We confirmed this with two orthogonal approaches: by performing long-read RNA-seq analysis to identify L1 transcripts that initiate in the L1 5′UTR and by H3K4me3 profiling to identify L1 promoters active in the human brain, benefiting from the fact that the signal of this histone modification spreads to the flanking (and thus unique) genomic context. We thus found bona fide evidence that full-length L1s are expressed in both the developing and the adult human brain. However, we acknowledge that with our approach, we miss the expression of polymorphic L1s not present in the reference genome. Future studies using individual-matched RNA-seq and long-read genome data will be crucial to investigate whether L1s individualize the neuronal transcriptome.

From our analysis, it is evident that not all L1 loci are expressed in the brain, but rather a small subset. Our data also indicate that the L1 integration site is important and that the presence of highly active nearby gene promoters or other regulatory elements is key for L1 expression. Thus, the activity of the surrounding genome is one parameter that is important for how this subset of L1s escapes silencing. In this respect, our results are similar to what have previously been found in cancer cell lines (*45*). In addition, single-nucleotide variants or small deletions in regulatory regions of individual L1 integrant could result in the avoidance of recruiting silencing factors. A previous study indicated that a subset of young L1s that have lost a Yin Yang 1 (YY1) binding site in the promoter avoids silencing in the brain in a DNA methylation–dependent manner (*32*). However, in our dataset, we found L1s both with and without the YY1 binding site to be expressed (fig. S6, A and B). Thus, we do not fully understand the mechanism by which these L1s escape silencing. However, it is worth noting that the adult brain tissue samples used for this study came from individuals aged between 69 and 87 years old at the time of death. It is well established that DNA methylation patterns change with age and there are emerging studies linking age-related epigenetic changes to activation of TE expression (*55*–*57*). This raises the possibility that some of the L1 transcripts we detect in adult neurons may be aging dependent. Future studies investigating the link between human brain aging and TE expression are needed to resolve this question. Another interesting aspect of our data is that L1HS elements appear to be globally silenced in brain development. This indicates that L1HS elements are controlled by unique, specialized mechanisms during brain development, likely to avoid abundant retrotransposition events in proliferating cell populations. The nature of this mechanism remains unknown, but it will be interesting to investigate further to understand how the human brain avoids waves of retrotransposition events during early development and what the consequences are if this mechanism fails.

The fact that many L1 promoters are active in the human brain demonstrates that L1s are a rich source of genetic sequences that provides a primate-specific layer of transcriptional complexity. Our data indicate that L1s influence the expression of protein-coding genes and noncoding transcripts in the human brain through several mechanisms, including acting as alternative promoters or by altering 5′UTR and 3′UTR. In addition, there is the possibility that L1-derived peptides or fusion peptides play important functional roles (*58*). One example of an L1-derived noncoding transcript that we identified is *LINC01876*, an L1-lncRNA that exploits the antisense promoter of an L1PA2 element that is transcriptionally active in human brain development. In the *LINC01876* promoter, the first amino acid of ORF0 is specifically mutated in humans, and the subsequent loss of ORF0 coding capacity correlates with the appearance of the L1-lncRNA. It is possible that this single-nucleotide variant, at a key position for the L1-life cycle, enables the escape of DNA methylation–mediated silencing resulting in transcription of the lncRNA.

Our loss-of-function studies of the L1-lncRNA *LINC01876* in cerebral organoids suggest that it may play an important role in regulating developmental timing during human brain development. *LINC01876* is a previously uncharacterized lncRNA, but we have noted that there is a T > C single-nucleotide polymorphism in the L1-derived promoter region of *LINC01876* that has been linked to major depressive disorders in a genome-wide association meta-analysis (*59*). Our data demonstrate that organoids in which *LINC01876* expression was silenced were smaller in size and displayed NPCs and neurons with a more mature transcriptome than control counterparts. These findings are reminiscent of previously observed differences when comparing human cerebral organoids to those derived from nonhuman great apes (*48*, *60*, *61*). Thus, our data provide experimental evidence as to how an L1 insertion may have contributed to the evolution of the human brain and provide a potential link between L1s and the genetics of neuropsychiatric disorders that will be interesting to study in more detail in the future.

In summary, our results illustrate how L1s provide a layer of transcriptional complexity in the brain and provide evidence for L1s as genetic elements with relevance in human brain function. It has been estimated that a new L1 germline insertion occurs in every 50 to 200 human births (*9*, *40*). This extensive L1 mobilization in the human population has resulted in hundreds of unfixed polymorphic L1 insertions in each human genome (*9*, *62*). Since L1s are highly polymorphic within the human population, the prevalence of certain L1 copies or single-nucleotide polymorphisms and structural variants in fixed L1s in the genome is therefore likely to influence the etiology of brain disorders. Thus, L1s represent a set of genetic materials that are implicated in the evolution of our brain and may contribute to important gene regulatory and transcriptional networks in the human brain. L1s should no longer be neglected, and these sequences need to be included in future investigations of the underlying genetic causes of human brain disorders.

## METHODS

### Human tissue

Human fetal forebrain tissue was obtained from material available following elective termination of pregnancy at the University Hospital in Malmö, Sweden, in accordance with the national ethical permit (Dnr 6.1.8-2887/2017). Postmortem cortical tissue was obtained in accordance with the national ethical permit (Dnr 2019-06582, beslut 2020-02-12). Written informed consent was obtained from all donors.

### Induced pluripotent stem cells

Human iPSC line generated by mRNA transfection was used: RBRC-HPS0328 606A1, hereafter referred to as HS1 (Riken, RRID:CVCL_DQ11). The iPSC line was maintained as previously described (*46*, *63*, *64*). Briefly, the iPSC lines were maintained on LN521 (0.7 μg/cm^2^; BioLamina)–coated Nunc multidishes in iPSC medium (StemMACS iPSC-Brew XF and 0.5% penicillin/streptomycin; Gibco) and were passaged 1:2 to 1:6 every 2 to 4 days once 70 to 90% confluency was reached. The medium was changed daily, and 10 μM Y27632 (Rock inhibitor, Miltenyi) was added when cells were passaged.

### Forebrain neural progenitor cells

iPSCs were differentiated into fbNPCs as previously described (*46*, *63*). Upon dissociation at 70 to 90% confluency, the cells were plated on LN111 (1.14 μg/cm^2^; BioLamina)–coated Nunc multidishes at a density of 10,000 cells/cm^2^ and grown in N2 medium [1:1 Dulbecco’s modified Eagle’s medium (DMEM)/F12 (21331020, Gibco) and Neurobasal (21103049, Gibco) supplemented with 1% N2 (Gibco), 2 mM l-glutamine (Gibco), and 0.2% penicillin/streptomycin]. SB431542 (10 μM; Axon) and noggin (100 ng/ml; Miltenyi) were given for dual SMAD inhibition. The medium was changed every 2 to 3 days. On day 9, N2 medium without dual SMAD inhibitors was used. On day 11, cells were dissociated and replated on LN111-coated Nunc multidishes at a density of 800,000 cells/cm^2^ in B27 medium [Neurobasal supplemented with 1% B27 without vitamin A (Gibco), 2 mM l-glutamine, and 0.2% penicillin/streptomycin Y27632 (10 μM), brain-derived neurotrophic factor (BDNF; 20 ng/ml; R&D), and l-ascorbic acid (0.2 mM; Sigma-Aldrich)]. Cells were kept in the same medium until day 14 when cells were harvested for downstream analysis.

### CRISPR interference

To silence the expression of *LINC01876* in iPSCs, we adapted a previously described protocol (*46*). Single-guide sequences were designed to recognize DNA regions near the TSS according to the GPP Portal (Broad Institute). The guide sequences were inserted into a dCas9-KRAB-T2A-GFP lentiviral backbone and pLV hU6-sgRNA hUbC-dCas9-KRAB-T2a-GFP, a gift from C. Gersbach (Addgene plasmid #71237, RRID:Addgene 71237), using annealed oligodendrocytes and the Bsm BI cloning site. Lentivirus was produced as described below, and iPSCs were transfected with multiplicity of infection of 10 of *LacZ* and *LINC01876*-targeting gRNA. Guide efficiency was validated using standard qRT-PCR techniques: *LINC01876* guide 1, ACGAGATTATAAGCCGCACC; *LINC01876* guide 2, AGGGGCGCCCGCCGTTGCCC; *LacZ*, TGCGAATACGCCCACGCGAT.

#### Green fluorescent protein–positive cell isolation of fbNPCs

At day 14, cells were detached with Accutase, resuspended in B27 medium containing RY27632 (10 μM) and Draq7 (1:1000; BD Biosciences), and strained with a 70-μm (BD Biosciences) filter. Gating parameters were determined by side and forward scatter to eliminate debris and aggregated cells. The green fluorescent protein (GFP)–positive gates were set using untransduced fbNPCs. The sorting gates and strategies were validated via reanalysis of sorted cells (>95% purity cutoff). A total of 200,000 GFP-positive/Draq7-negative cells were collected per sample, spun down at 400*g* for 5 min, and snap-frozen on dry ice. Cell pellets were kept at −80°C until RNA was isolated.

#### GFP-positive cell isolation of transduced iPSCs

Seven days after transduction, cells were detached with Accutase, resuspended in iPSC medium containing RY27632 (10 μM) and Draq7 (1:1000), and strained with a 70-μm filter. Gating parameters were determined by side and forward scatter to eliminate debris and aggregated cells. The GFP-positive gates were set using untransduced iPSCs. The sorting gates and strategies were validated via reanalysis of sorted cells (>95% purity cutoff). A total of 200.000 GFP-positive/Draq7-negative cells were collected per sample, spun down at 400*g* for 5 min and resuspended in iPSC medium containing RY27632 (10 μM) and expanded as described above and frozen down for further use. Detailed protocol can be found at DOI: dx.doi.org/10.17504/protocols.io.yxmvm25n9g3p/v1.

### Lentiviral production

Lentiviral vectors were produced according to Zufferey *et al.* (*65*) and were titered by qRT-PCR. Briefly, human embryonic kidney–293T cells (RRID:CVCL_0063) were grown to a confluency of 70 to 90% for lentiviral production. Third-generation packaging and envelope vectors [pMDL (#12251, Addgene), psRev (#12253, Addgene), and pMD2G (#12259, Addgene)] together with polyethyleneimine (PN 23966, PEI Polysciences) in Dulbecco’s phosphate-buffered saline (DPBS; Gibco) were used in conjunction with the lentiviral plasmids previously generated. The lentivirus was harvested 2 days after transfection. The medium was collected, filtered, and centrifuged at 25,000*g* for 1.5 hours at 4°C. The supernatant was removed from the tubes, and the virus was resuspended in DPBS and left at 4°C. The resulting lentivirus was aliquoted and stored at −80°C.

### Quantitative real-time polymerase chain reaction

Total RNA was first extracted using the miniRNeasy kit (QIAGEN). Complementary DNA (cDNA) was generated using the Maxima First Strand cDNA Synthesis Kit (Thermo Fisher Scientific). Quantitative PCR was performed using SYBR Green I master (Roche) on a LightCycler 480 (Roche). The 2^−ΔΔ*C*t^ method was used to normalize expression to control, relative to glyceraldehyde-3-phosphate dehydrogenase (GAPDH) and B-ACTIN as described previously (*66*).The gene primers used are as follows: *LINC01876*, 5′-AATCCGTGCCAGCAGTAAGT-3′ (forward) and 5′-GGACCTCTTCAAGTCCCAGG-3′ (reverse); *ACTB*, 5′-CCTTGCACATGCCGGAG-3′ (forward) and 5′-GCACAGAGCCTCGCCTT-3′; *GAPDH*, 5′-TTGAGGTCAARGAAGGGGTC-3′ (forward) and 5′-GAAGGTGAAGGTCGGAGTCA-3′ (reverse).

### Human cerebral organoid culture

To generate the human cerebral-like organoids, we followed the protocol detailed in (*46*). We used three HS1-derived lines obtained by transduction and FACS sorting as described above: one control line (guide against *LacZ*) and two *LINC01876* CRISPRi lines (guide 1 and guide 2). Briefly, 8000 cells per well were plated in a 96-well plate (Costar, ultra low attachment, round bottom; REF 7007) with 250 μl of mTeSR1 (STEMCELL Technologies Inc.) and 10 μM RY27632. This is considered day −5 of the differentiation of the iPSC-derived human forebrain organoids. On days −3 and −1, the medium was changed (150 and 200 μl of mTeSR1, respectively). At day 0, the cells are fed with neural induction medium [DMEM/F12 medium, N2 supplement (1:100), l-glutamine (2 mM), penicillin/streptomycin (1:500), nonessential amino acids (1:100), and heparin (2 μg/ml)] enriched with 3% knockout replacement serum (#10828010, Gibco). On days 2, 4, and 6, the organoids were fed with neural induction medium with no added knockout replacement serum.

On day 8, the organoids were embedded in 30 to 50 μl of Matrigel (Corning) and incubated at 37°C for 25 min to allow the Matrigel to solidify. The organoids were then transferred in Corning (REF 3471) six-well plates with flat bottoms containing 4 ml per well of cortical differentiation medium [F12 medium (−glut) (48.5%), Neurobasal (48.5%), N2 supplement (1:200), B27 supplement (−vitamin A; 1:100), l-glutamine (2 mM), penicillin/streptomycin (1:500), nonessential amino acids (1:200), β-mercaptoethanol (50 μM), and insulin (2.5 μg/ml)].

On days 10 and 12 of the differentiation, the medium was changed exchanging 3 ml per well for 3 ml of fresh cortical differentiation medium. On days 15, 17, 19, 21, and 23, ∼4 ml of the medium was replaced with 4 ml of improved differentiation medium + A [F12 media (−glut) (48.5%), Neurobasal (48.5%), N2 supplement (1:200), B27 supplement (+vitamin A; 1:50), l-glutamine (2 mM), penicillin/streptomycin (1:500), nonessential amino acids (1:200), β-mercaptoethanol (50 μM), insulin (2.5 μg/ml), and ascorbic acid (400 μM)]. From day 25, the medium was changed every 3 days with 3 to 4 ml of cortical terminal differentiation medium [F12 media (−glut) (48.5%), Neurobasal (48.5%), N2 supplement (1:200), 800 μL of B27 supplement (+vitamin A; 1:50), l-glutamine (2 mM), penicillin/streptomycin (1:500), nonessential amino acids (1:200), β-mercaptoethanol (50 μM), insulin (2.5 μg/ml), ascorbic acid (400 μM), BDNF (10 ng/μl), adenosine 3′,5′-monophosphate (200 μM), and glial cell line–derived neurotrophic factor (10 ng/μl)].

We performed three independent replicates of the CRISPRi experiment in cerebral organoids (three batches). We measured the size of 10 organoids per time point and condition in each batch. All the diameter measurements of the organoids were taken with the measure tool from ImageJ (RRID:SCR_003070). The chosen measuring unit was micrometers.

The statistical analysis to test the difference in organoid growth upon L1-lncRNA CRISPRi per guide was performed using a two-way analysis of variance (ANOVA), adjusting for multiple comparison using a Dunnett correction. The statistical analysis to test the difference in organoid growth pooling both gRNAs for the L1-lncRNA CRISPRi was performed using a mixed-effects analysis and a Sidak correction for multiple comparisons. Detailed can be found at DOI: dx.doi.org/10.17504/protocols.io.e6nvwjo27lmk/v1.

### Immunocytochemistry

The cells were washed three times with DPBS and fixed for 10 min with 4% paraformaldehyde (Merck Millipore), followed by three more rinses with DPBS. The fixed cells were then blocked for 60 min in a blocking solution of KPBS with 0.25% Triton X-100 (Thermo Fisher Scientific) and 5% donkey serum at room temperature.

The primary antibody [rabbit anti-FOXG1 (Abcam, RRID: AB_732415); 1:50] was added to the blocking solution and incubated overnight at room temperature. Subsequently, the cells were washed three times with KPBS. The secondary antibody [donkey anti-rabbit Cy3 (catalog no. 711165152, Jackson ImmunoResearch, RRID:AB_2307443); 1:200] was added with 4′,6-diamidino-2-phenylindole (DAPI) (Sigma-Aldrich; 1:1000) to the blocking solution and incubated at room temperature for 1 hour, followed by two to three rinses with KPBS. The cells were visualized on a Leica microscope (model DMI6000 B). Detail protocol can be found at DOI: dx.doi.org/10.17504/protocols.io.5qpvor7pdv4o/v1.

### Immunohistochemistry

Organoids were fixed in 4% paraformaldehyde for 2 hours at room temperature. They were subsequently washed three times with KPBS and left in a 1:1 30% sucrose solution and OCT (catalog no. 45830, HistoLab) mixture overnight at 4°C. Organoids were then transferred to a cryomold containing OCT, frozen on dry ice, and stored at −80°C in freezer bags.

Before staining, organoids were sectioned on a cryostat at −20°C at a thickness of 20 μm and placed onto Superfrost plus microscope slides. They were then washed three times with KPBS for 5 min and subsequently blocked and permeabilized in 0.1% Triton X-100 and 5% normal donkey serum in KPBS for 1 hour at room temperature. The primary antibody [rabbit anti-PAX6 (catalog no. 901301, BioLegend, RRID:AB_2565003), 1:300 dilution; and rat anti-ZO1 (catalog no. NB110-68140, Novus, RRID:AB_1111431), 1:300 dilution] was added to the blocking solution and incubated overnight at room temperature. Subsequently, the sections were washed three times with KPBS. The secondary antibody [donkey anti-rabbit Cy3 (catalog no. 711165152, Jackson ImmunoResearch, RRID:AB_2307443), 1:200; and donkey anti-rat Cy5 (catalog no. 712175153, Jackson ImmunoResearch, RRID: AB_2340672), 1:200] was added with DAPI (Sigma-Aldrich; 1:1000) to the blocking solution and incubated at room temperature for 1 hour, followed by two to three rinses with KPBS. Sections were imaged using Operetta CLS (PerkinElmer). Detail protocol can be found at DOI: dx.doi.org/10.17504/protocols.io.n92ldp22nl5b/v1.

### Single-nucleus isolation

The nucleus isolation from the embryonic brain tissue and organoids was performed as described previously (*36*). Briefly, the tissue and organoids were thawed and dissociated in ice-cold lysis buffer [0.32 M sucrose, 5 mM CaCl_2_, 3 mM MgAc, 0.1 mM Na_2_ EDTA, 10 mM tris-HCl (pH 8.0), and 1 mM dithiothreitol] using a 1-ml tissue douncer (Wheaton). The homogenate was carefully layered on top of a sucrose cushion [1.8 M sucrose, 3 mM MgAc, 10 mM tris-HCl (pH 8.0), and 1 mM dithiothreitol] before centrifugation at 30,000*g* for 2 hours and 15 min. Pelleted nuclei were softened for 10 min in 100 μl of nuclear storage buffer [15% sucrose, 10 mM tris-HCl (pH 7.2), 70 mM KCl, and 2 mM MgCl_2_] before being resuspended in 300 μl of dilution buffer [10 mM tris-HCl (pH 7.2), 70 mM KCl, and 2 mM MgCl_2_] and run through a cell strainer (70 μm). Cells were run through the FACS (FACS Aria, BD Biosciences) at 4°C at a low flow rate using a 100-μm nozzle (reanalysis showed >99% purity). Nuclei intended for bulk RNA-seq were pelleted at 1300*g* for 15 min. Detail protocol can be found DOI: dx.doi.org/10.17504/protocols.io.5jyl8j678g2w/v1.

### 3′ and 5′ single-nucleus sequencing

Nuclei or cells intended for single-cell/nucleus RNA-seq (8500 nuclei/cells per sample) were directly loaded onto the Chromium Next GEM Chip G or Chromium Next GEM Chip K Single Cell Kit along with the reverse transcription mastermix following the manufacturer’s protocol for the Chromium Next GEM single cell 3′ kit (PN-1000268, 10x Genomics) or Chromium Next GEM Single Cell 5’ Kit (PN-1000263, 10x Genomics), respectively, to generate single-cell gel beads in emulsion. cDNA amplification was done as per the guidelines from 10x Genomics using 13 cycles of amplification for 3′ and 15 cycles of amplification for 5′ libraries. Sequencing libraries were generated with unique dual indices (TT set A) and pooled for sequencing on a Novaseq6000 using a 100-cycle kit and 28-10-10-90 reads.

#### Single-cell/nucleus RNA-seq analysis

##### Gene quantification

The raw base calls were demultiplexed and converted to sample-specific fastq files using 10x Genomics Cell Ranger mkfastq (version 3.1.0; RRID:SCR_017344) (*67*). Cell Ranger count was run with default settings, using an mRNA reference for single-cell samples and a pre-mRNA reference (generated using 10x Genomics Cell Ranger 3.1.0 guidelines) for single-nucleus samples.

To produce velocity plots, loom files were generated using velocyto (*43*) (version 0.17.17; RRID:SCR_018167) run 10× in default parameters, masking for TEs [same general feature format (GTF) file as input for TEtranscripts; see the “TE subfamily quantification” section] and GENCODE version 36 as guide for features. Plots were generated using velocyto.R (see GitHub under src/analysis/fetal_velocity.Rmd).

##### Clustering

Samples were analyzed using Seurat (version 3.1.5; RRID:SCR_007322) (*68*). For each sample, cells were filtered out if the percentage of mitochondrial content was over 10% (perc_mitochondrial). For adult samples, cells were discarded if the number of detected features (nFeature_RNA) was higher than two SDs over the mean in the sample (to avoid keeping doublets) or lower than an SD below the mean in the sample (to avoid low quality cells). For fetal samples, cells were discarded if the number of detected features was higher than two SDs over the mean in the sample or lower than 2000 features detected. Counts were normalized using the centered log ratio transformation (Seurat::NormalizeData), and clusters were found with a resolution of 0.5 (Seurat::FindClusters).

##### Gene differential expression analysis

We used Seurat’s FindMarkers grouped by cell types and on default parameters as for version 4.3.0 to identify differentially expressed genes (Wilcoxon test). A gene was considered to be differentially expressed on a cell type if its adjusted *P* value was below 0.05 and its average log_2_FoldChange is over 0.25 (default).

##### TE quantification

We used an in-house pseudo-bulk approach to processing snRNA-seq data to quantify TE expression per cluster, similar to what has been previously described (*36*). All clustering, normalization and merging of samples were performed using the contained scripts of get_clusters.R [get_custers() from the Sample class] and merge_samples.R [merge_samples() from the Experiment class] of trusTEr (version 0.1.1; doi:10.5281/zenodo.7589548). Documentation of the pipeline can be found at https://raquelgarza.github.io/truster/.

The main functionality of trusTEr is to create collections of reads to remap and quantify TE subfamilies or elements per group of cells. The function tsv_to_bam() backtraces cells barcodes to Cell Ranger’s output binary alignment map (BAM) file. tsv_to_bam() runs using subset-bam from 10x Genomics version 1.0 (RRID:SCR_023216). As the next step of the pipeline, the function filter_UMIs() filters potential PCR duplicates in the BAM files; this step uses Pysam version 0.15.1 (RRID:SCR_021017). Next, to convert BAM to fastq files, we used bamtofastq from 10x Genomics (version 1.2.0; RRID: SCR_023215). The remapping of the clusters was performed using STAR aligner (version 2.7.8a; RRID:SCR_004463). Quantification of TE subfamilies was done using TEcount (version 2.0.3; RRID:SCR_023208), and individual elements were quantified using featureCounts (subread version 1.6.3; RRID:SCR_012919). The normalization step of trusTEr, to integrate with Seurat and normalize TE subfamilies’ expression, was performed using Seurat version 3.1.5 (RRID:SCR_007322).

For the purposes of this paper, we combined the samples from the same condition (all embryonic samples and all adult samples). The quantification was run twice: with all samples together and per sample in the combined clustering. For the fetal samples, we also ran trusTEr grouping clusters per cell cycle state, for which we prepared a directory with tsv files containing the barcodes of the cells in each of the clusters of interest (e.g., cluster0_cycling.tsv, cluster0_noncycling.tsv, …) and ran the set_merge_samples_outdir function from the Experiment class to register these as cluster objects.

### Bulk RNA-seq

Total RNA was isolated from nuclei, cell culture samples, or tissue using the RNeasy Mini Kit (QIAGEN). Libraries were generated using Illumina TruSeq Stranded mRNA library prep kit [poly(A) selection] and sequenced on a NextSeq500 (PE, 2 × 150 bp). Protocol can be found at DOI: https://dx.doi.org/10.17504/protocols.io.36wgqjqbkvk5/v1.

#### Bulk RNA-seq analysis

##### TE subfamily quantification

For the quantification of TE subfamilies, the reads were mapped using STAR aligner (version 2.6.0c; RRID:SCR_004463) (*69*) with an hg38 index and GENCODE version 36 as the guide GTF (--sjdbGTFfile), allowing for a maximum of 100 multimapping loci (--outFilterMultimapNmax 100) and 200 anchors (--winAnchorMultimapNmax). The rest of the parameters affecting the mapping was left in default as for version 2.6.0c.

The TE subfamily quantification was performed using TEcount from the TEToolkit (version 2.0.3; RRID:SCR_023208) in mode multi (--mode). GENCODE annotation v36 was used as the input gene GTF (--GTF), and the provided hg38 GTF file from the author’s web server was used as the TE GTF (--TE) (*35*).

##### TE quantification

Reads were mapped using STAR aligner (version 2.6.0c; RRID:SCR_004463) (*69*) with an hg38 index and GENCODE version 30 (adult data) and 36 (fetal data) as the guide GTF (--sjdbGTFfile). To quantify only confident alignments, we allowed a single mapping locus (--outFilterMultimapNmax 1) and a ratio of mismatches to the mapped length of 0.03 (--outFilterMismatchNoverLmax).

To measure the antisense transcription over a feature, we divided the resulting BAM file into two, containing the forward and reverse transcription, respectively. We used SAMtools view (version 1.9; RRID:SCR_002105) (*70*) to keep only the alignments in forward transcription, we separated alignments of the second pair mate if they mapped to the forward strand (-f 128 -F 16) and alignments of the first pair mate if they map to the reverse strand (-f 80). To keep the reverse transcription, we kept alignments of the second pair mate if they mapped to the reverse strand (-f 144) and alignments of the first pair mate if they mapped to the forward strand (-f 64 -F 16).

Both BAM files were then quantified using featureCounts from the subread package (version 1.6.3; RRID:SCR_012919) (*71*) forcing strandness to the features being quantified (-s 2). For consistency (and to avoid quantifying over simple repeats, small RNAs, and low-complexity regions), we input the same curated hg38 GTF file provided by the TEtranscripts authors (*35*).

##### Gene quantification

Reads were mapped using STAR aligner (version 2.6.0c; RRID:SCR_004463) (*69*) with an hg38 index and GENCODE version 36 as the guide GTF (--sjdbGTFfile), and no other parameters were modified (default values for --outFilterMultimapNmax, --outFilterMismatchNoverLmax, and --winAnchorMultimapNmax). Genes were quantified using featureCounts from the subread package (version 1.6.3; RRID:SCR_012919) (*71*) forcing strandness (-s 2) to quantify by gene_id (-g) from the GTF of GENCODE version 36.

##### Differential gene expression analysis

We performed differential expression analysis using DESeq2 (version 1.28.1; RRID:SCR_015687) (*72*) with the read count matrix from featureCounts (subread version 1.6.3; RRID:SCR_012919) as input. Fold changes were shrunk using DESeq2:: lfcShrink.

For the produced heatmaps, counts were normalized by median of ratios as described by Love *et al.* (*72*), summed with a pseudo-count of 0.5 and log_2_-transformed.

For further detail, please refer to the Rmarkdown on the GitHub.

##### Transcript assembly and quantification

Transcript assembly for each of the short-read bulk RNA-seq samples was performed using StringTie (version 1.3.3b; RRID:SCR_016323) (*73*) with GENCODE hg38 version 38 as guide (-G). Output assemblies were merged by StringTie -merge, using the same GENCODE annotation as guide (-G). Transcript assemblies were then performed for each sample using the resulting GTF output from StringTie merge as the guide reference annotation (-G); the resulting GTFs from this step will hereon be referred as the samples’ transcript assembly GTF. Read count tables were then generated using the accessory script from StringTie prepDE.py (https://github.com/gpertea/stringtie/blob/master/prepDE.py).

To identify L1 chimeras (table S3), we concatenated the samples’ transcript assembly GTFs. We kept only unique transcript features with over 1 kbp of length. We created an auxiliary GTF file keeping only the TSS of each transcript plus 100-bp windows in both directions—this file will hereon be referred to as the transcripts’ TSS GTF. Using BEDTools intersect and forcing for opposing strands (-S), we intersected the transcripts’ TSS GTF to full-length (>6 kbp) L1PAs using the RepeatMasker’s annotation (open-4.0.5).

Transcripts’ read count matrices were normalized using the DESeq2 (version 1.28.1; RRID:SCR_015687) (*72*) sizeFactors as calculated using the gene count matrix (see the “Differential gene expression analysis” section). A transcript was considered to be expressed on a sample if its normalized expression value exceeded that of 20. These transcripts were considered for Venn diagrams shown in Fig. 4E.

##### Differential TE subfamilies expression analysis

We performed differential expression analysis using DESeq2 (version 1.28.1; RRID:SCR_015687) (*72*) with the read count matrix from TEcount (version 2.0.3; RRID:SCR_023208) (*35*) using only the TE subfamilies entries. Fold changes were shrunk using DESeq2:: lfcShrink.

Using the gene DESeq2 object (see section above), we normalized the TE subfamily counts by dividing the read count matrix by the sample distances (sizeFactor) as calculated by DESeq2 with the quantification of genes without multimapping reads (see the “Bulk RNA-seq analysis: Gene quantification” section). For heatmap visualization, a pseudo-count of 0.5 was added and log_2_-transformed.

##### Comparison between sense and antisense transcription over TEs

To normalize uniquely mapped read counts per strand (see the “Bulk RNA-seq analysis: TE quantification” section), we divided the read count matrix by the sample distances (sizeFactor) as calculated by DESeq2 (version 1.28.1; RRID:SCR_015687) with the quantification of genes without multimapping reads (see the “Bulk RNA-seq analysis: Gene quantification” section).

Each point in the boxplot (Figs. 1E and 4E) refers to a sample. “Antisense” refers to counts of reverse transcription in forward features and counts from forward transcription in reverse features. “Sense” refers to counts of reverse transcription in features annotated in the reverse strand and forward counts in features annotated in the forward strand. Boxplots were produced by summing counts of the same subfamily and strand, per sample, per the direction of transcription (e.g., all L1PA2s in the reverse strand were summed using only the counts from the reverse strand).

##### Comparing the ratio of detected elements of all L1s

Once normalized for the counts of individual elements by the gene sizeFactors (see the “Comparison between sense and antisense transcription over TEs” section; Figs. 1F and 3C), we defined a “detected” element as an element with a mean of >10 normalized counts in the group of samples of interest. The total number of elements is the number of elements from a particular subfamily annotated in the GTF file that was input to featureCounts (version 1.6.3; RRID:SCR_012919).

##### Transcription over evolutionary young L1 elements in bulk datasets

The browser extensible data (BED) file version of TEcount’s GTF file was used to create BED files containing all L1HS, L1PA2, L1PA3, and L1PA4 elements longer than 6 kbp (full length). These BED files were then split by the strand of the element.

Using the bigwig files of the uniquely mapped BAM files, we created four matrices per dataset using the DeepTools’ (version 2.5.4; RRID:SCR_016366) computeMatrix function (*74*)—one for elements annotated in the positive strand using only the bigwig files with forward transcription (transcription in sense of the element), another one for elements annotated in the reverse strand using only bigwig files with reverse transcription (transcription in sense of the element), and another two with the antisense transcription being used (e.g., elements annotated in the positive strand using reverse transcription bigwig files). We then concatenate the matrices of transcription in sense of the elements together using rbind from computeMatrixOperations (*74*). The same operation was performed for the antisense matrices. Heatmaps were plotted using plotHeatmap (*74*), setting missing values to white (--missingDataColor white), and colorMap to blues (sense) or reds (antisense).

To investigate whether the expressed elements contained an intact YY1 binding site, we extracted the relevant sequences using getfasta from BEDTools (version 2.30.0; RRID:SCR_006646) (*75*) using GRCh38.p13 as input fasta (-fi) and forcing strandness (-s). We quantified the number of elements with an exact match to the YY1 binding motif (CAAGATGGCCG) (*76*) in the first 100 bp of the element (see GitHub under src/analysis/yy1_present.py).

### PacBio Iso-Seq sample preparation

Total RNA was obtained from tissue samples using miRNA Easy Mini Kit (QIAGEN). RNA samples were subsequently put on dry ice and shipped to the National Genomics Infrastructure of Sweden. There, input quality control of samples was performed on the Agilent Bioanalyzer instrument, using the Eukaryote Total RNA Nano kit (Agilent) to evaluate RNA Integrity Number (RIN) and concentration. The sample libraries were prepared as described in “Procedure & Checklist—Iso-Seq Express Template Preparation for Sequel and Sequel II Systems” (PN-101763800, PacBio, version 02; October 2019) using the NEBNext Single Cell/Low Input cDNA Synthesis & Amplification Module (catalog nos. E6421S for 24 reactions and E6421L for 96 reactions, New England Biolabs), the Iso-Seq Express Oligo Kit (catalog no. PN-101737500, PacBio), ProNex beads [catalog nos. NG2001 (10 ml), NG2002 (125 ml), and NG2003 (500 ml), Promega] and the SMRTbell Express Template Prep Kit 2.0 (catalog no. PN-100938900, PacBio). Total RNA (300 ng) was used for cDNA synthesis, followed by 12 + 3 cycles of cDNA amplification. In the purification step of amplified cDNA, the standard workflow was applied (sample is composed primarily of transcripts centered around 2 kb). After purification, the amplified cDNA went into the SMRTbell library construction. Quality control of the SMRTbell libraries was performed with the Qubit dsDNA HS kit (catalog no. Q32851, Invitrogen) and the Agilent Bioanalyzer High Sensitivity Kit. Primer annealing and polymerase binding were performed using the Sequel II binding kit 2.0 (catalog no. PN-101789500, PacBio). Last, the samples were sequenced on Sequel II and Sequel IIe System using Sequel II Sequencing Plate 2.0, with an on-plate loading concentration of 110 pM, a movie time of 24 hours, and a pre-extension time of 2 hours.

Detail protocol can be found at DOI: dx.doi.org/10.17504/protocols.io.yxmvm25j6g3p/v1. For additional information, please contact the National Genomics Infrastructure of Sweden.

#### Iso-Seq mapping to L1HS/PA2 consensus sequence

A L1HS and L1PA2 consensus sequence was used to create a minimap2 (version 2.24; RRID:SCR_018550) (*77*) index (minimap2 -d L1consensus.mmi L1consensus.fa) to map full-length nonconcatemer reads (HiFi reads). The density of mapped reads was visualized in the Integrative Genomics Viewer (version 2.12.3; RRID:SCR_011793) (*78*). The number of mapped reads in the L1s 5′UTR was retrieved using SAMtools view (-c) (version 1.9; RRID:SCR_002105), specifying the first 900 bp of the consensus sequence as the coordinates of interest.

### Isolation of NeuN^+^ cells

Nuclei were isolated from frozen tissue as described above. Before FACSing, nuclei were incubated with recombinant Alexa Fluor 488 anti-NeuN antibody [EPR12763]–neuronal marker (catalog no. ab190195, Abcam, RRID:AB_2716282) at a concentration of 1:500 for 30 min on ice as previously described (*79*). The nuclei were run through the FACS at 4°C with a low flow rate using a 100-mm nozzle, and 300,000 Alexa Fluor 488–positive nuclei were sorted. The sorted nuclei were pelleted at 1300*g* for 15 min and resuspended in 1 ml of ice-cold nuclear wash buffer (20 mM Hepes, 150 mM NaCl, 0.5 mM spermidine, 1× cOmplete protease inhibitors, 0.1% bovine serum albumin) and 10 μl per antibody treatment of ConA-coated magnetic beads (Epicypher) added with gentle vortexing (pipette tips for transferring nuclei were precoated with 1% bovine serum albumin). Protocol can be found at DOI: dx.doi.org/10.17504/protocols.io.4r3l27pejg1y/v1.

### CUT&RUN

We followed the protocol detailed by the Henikoff laboratory (*41*). Briefly, 100,000 sorted nuclei were washed twice [20 mM Hepes (pH 7.5), 150 mM NaCl, 0.5 mM spermidine, and 1× Roche cOmplete protease inhibitors] and attached to 10 ConA-coated magnetic beads (Bangs Laboratories) that had been preactivated in binding buffer [20 mM Hepes (pH 7.9), 10 mM KCl, 1 mM CaCl_2_, and 1 mM MnCl_2_]. Bead-bound cells were resuspended in 50 μl of buffer [20 mM Hepes (pH 7.5), 0.15 M NaCl, 0.5 mM spermidine, 1× Roche cOmplete protease inhibitors, 0.02% (w/v) digitonin, and 2 mM EDTA] containing primary antibody (rabbit anti-H3K4me3: 39159, Active Motif, RRID:AB_2615077; or goat anti-rabbit immunoglobulin G: ab97047, Abcam, RRID:AB_10681025) at 1:50 dilution and incubated at 4°C overnight with gentle shaking. Beads were washed thoroughly with digitonin buffer [20 mM Hepes (pH 7.5), 150 mM NaCl, 0.5 mM spermidine, 1× Roche cOmplete protease inhibitors, and 0.02% digitonin]. After the final wash, pA-MNase (a gift from S. Henikoff) was added to the digitonin buffer and incubated with the cells at 4°C for 1 hour. Bead-bound cells were washed twice, resuspended in 100 μl of digitonin buffer, and chilled to 0° to 2°C. Genome cleavage was stimulated by the addition of 2 mM CaCl_2_ at 0°C for 30 min. The reaction was quenched by the addition of 100 μl of 2× stop buffer [0.35 M NaCl, 20 mM EDTA, 4 mM EGTA, 0.02% digitonin, glycogen (50 ng/μl), ribonuclease A (50 ng/μl), yeast spike-in DNA (10 fg/μl; a gift from S. Henikoff)] and vortexing. After 10 min of incubation at 37°C to release genomic fragments, cells and beads were pelleted by centrifugation (16,000*g* for 5 min at 4°C), and fragments from the supernatant were purified. Illumina sequencing libraries were prepared using the HyperPrep Kit (KAPA) (catalog no. 7962347001, Roche) with unique dual-indexed adapters (KAPA) (catalog no. 8278555702, Roche), pooled, and sequenced on a NextSeq500 instrument (Illumina). Detail protocol can be found at DOI: dx.doi.org/10.17504/protocols.io.j8nlkwb8dl5r/v1.

#### CUT&RUN analysis

Paired-end reads (2 × 75) were aligned to the human genome (hg38) using bowtie2 (version 2.3.4.2; RRID:SCR_016368) (*80*) (–local –very-sensitive-local –no-mixed –no-discordant –phred33 -I 10 -X 700), converted to BAM files with SAMtools (version 1.4; RRID:SCR_002105) and sorted (SAMtools version 1.9; RRID:SCR_002105). Reads per kilobase per million mapped reads (RPKM) normalized bigwig coverage tracks were made with bamCoverage (DeepTools, version 2.5.4; RRID:SCR_016366) (*74*).

Tag directories were created using Homer (version 4.10; RRID:SCR_010881) (*81*) makeTagDirectory on default parameters. Peak calling was performed using findPeaks (Homer), using the option histone as style (-style). The rest of the parameters were left on default options. Peaks were then annotated using the script annotatePeaks.pl (Homer; http://homer.ucsd.edu/homer/ngs/annotation.html) and intersected (BEDtools, version 2.30.0; RRID:SCR_006646) to bed files containing coordinates of >6-kbp L1HS, L1PA2, L1PA3, or L1PA4. Matrices for heatmaps were created (computeMatrix, DeepTools, version 2.5.4; RRID:SCR_016366) using the peaks with an overlap on these elements (only peaks that were called in all samples of a dataset) and visualized using plotHeatmap (DeepTools).

## Supplementary Materials

### This PDF file includes:

Figs. S1 to S6 Tables S1 to S3 References
