# Supplementary material for: LINE-1 retrotransposons drive human neuronal transcriptome complexity and functional diversification
Source: Sci Adv. 2023 Nov 1;9(44):eadh9543. doi: 10.1126/sciadv.adh9543 (PMC10619931; doi:10.1126/sciadv.adh9543)
Supplement: Supplementary file 1 — Figs. S1 to S6 Tables S1 to S3 References [file sciadv.adh9543_sm.pdf]

Science Advances
AAAS

Supplementary Materials for

LINE-1 retrotransposons drive human neuronal transcriptome complexity and functional diversification

Raquel Garza *et al.*

Corresponding author: Johan Jakobsson, johan.jakobsson@med.lu.se

Sci. Adv. **9**, eadh9543 (2023)
DOI: 10.1126/sciadv.adh9543

This PDF file includes:

Figs. S1 to S6
Tables S1 to S3
References

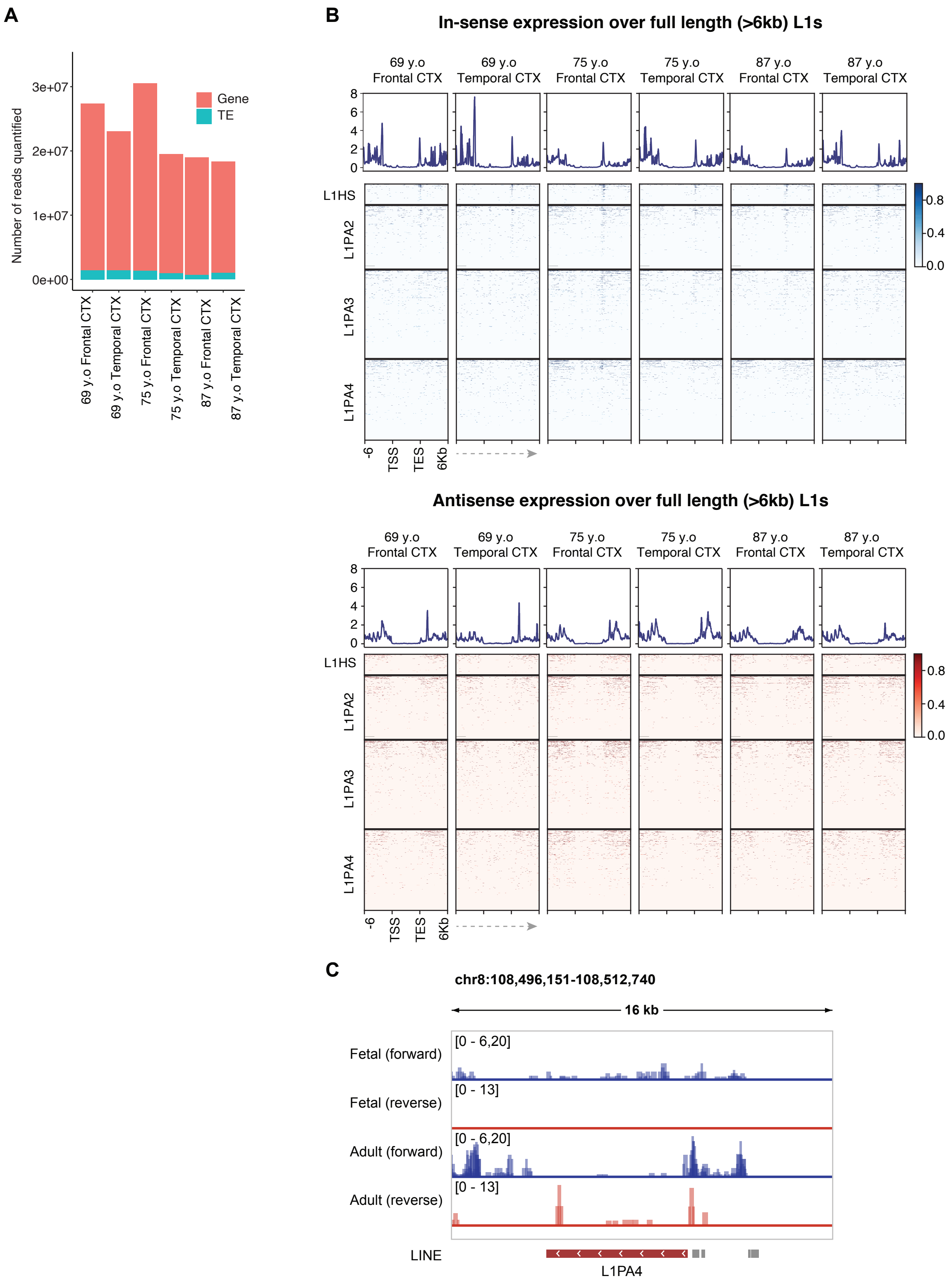

Figure S1 Quality control for the validation of L1 expression in the adult human brain. A) Number of reads quantified as genes or TEs per sample, as quantified by TEcounts. B) Expression (RPKM) over full length (>6kbp) L1HS, L1PA2, L1PA3 and L1PA4, plus 6kbp flanking regions. Blue heatmaps showing the signal per sample in sense of the annotated element. Red heatmaps showing signal in antisense. C) Genome browser tracks showing an adult-specific expression of a >6kbp L1PA4 with antisense transcription initiated in its promoter. Transcription is split by strand (blue = forward; red = reverse).

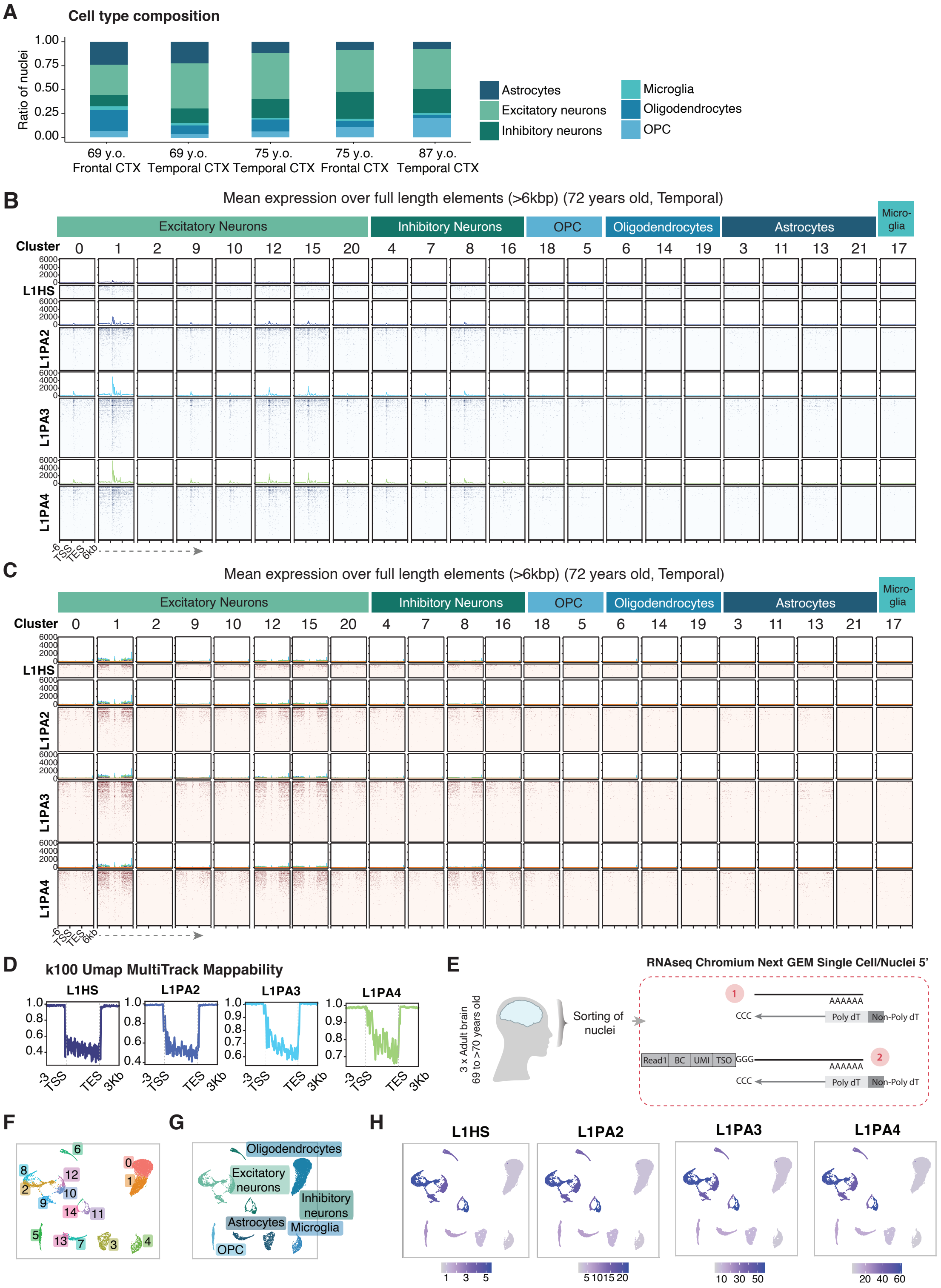

Figure S2 L1 expression in neurons in the adult human brain. A) Cell type composition in the snRNA-seq of adult samples. B) Expression (RPKM) over full length (>6kbp) L1HS, L1PA2, L1PA3 and L1PA4, plus 6kbp flanking regions in each cluster for one of the adult samples. Blue heatmaps showing the signal per cluster in sense of the annotated element. Red heatmaps showing signal in antisense. Top annotation indicates the cell type of the cluster in question. D) Single-read mappability score for full-length (>6kbp) young L1 subfamilies (read length of 100) as reported for hg38 by Karimzadeh, et al. 2018 *(86)* (tracks available at UCSC table browser)). E) Schematic of 5' enrichment Chromium Next GEM library. F) snRNA-seq UMAP colored by cluster. G) UMAP colored by characterized cell types. H) Pseudo-bulk cluster expression of young L1 subfamilies on UMAP.

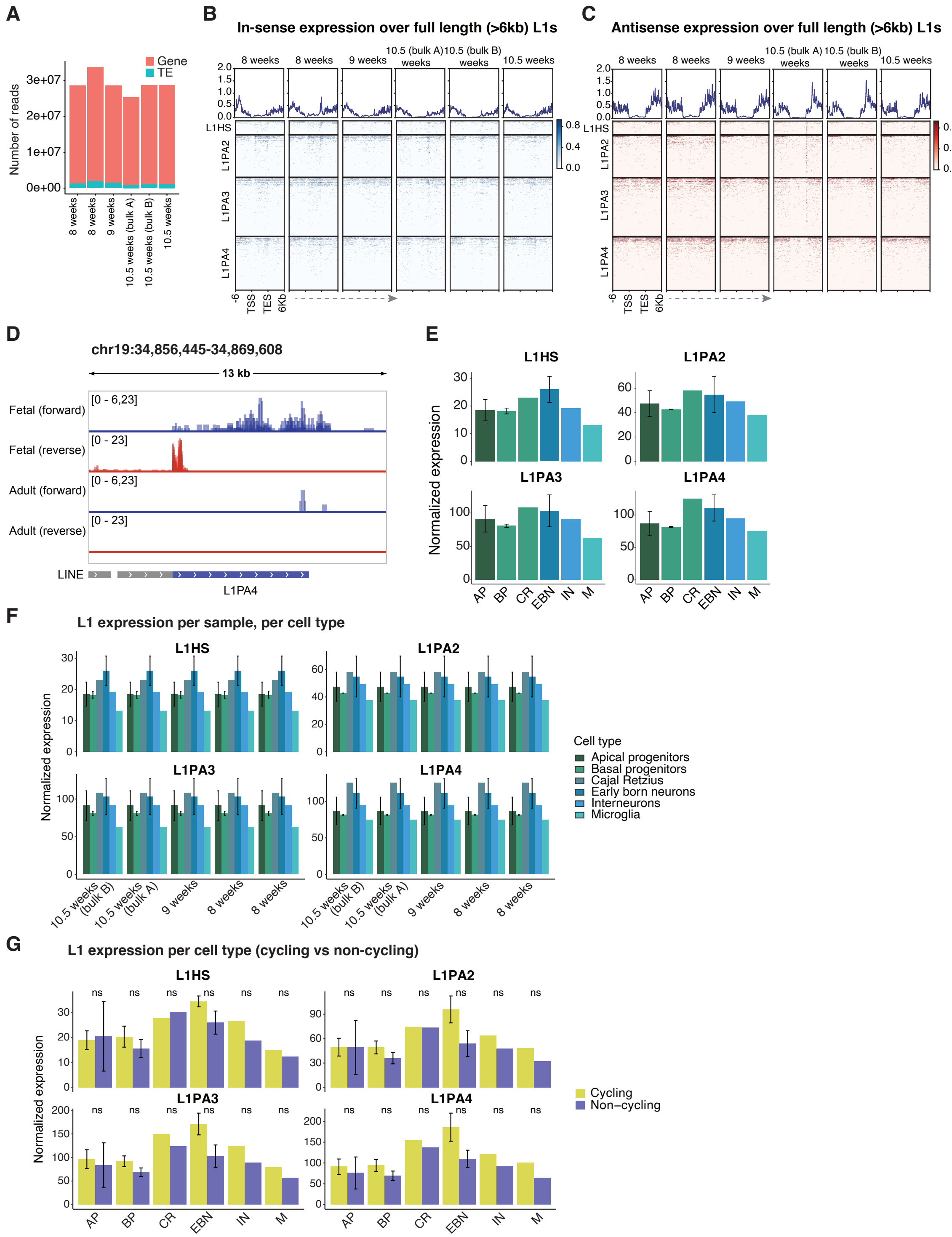

Figure S3 Quality control for the validation of L1 expression in bulk and different cell types of the fetal forebrain. A) Number of reads quantified as genes or TEs per sample, as quantified by TEcounts. B) Expression (RPKM) over full length (>6kbp) L1HS, L1PA2, L1PA3 and L1PA4, plus 6kbp flanking regions. Blue heatmaps showing the signal per sample in sense of the annotated element. C) Red heatmaps showing signal in antisense D) Genome browser tracks showing fetal-specific expression of a >6kbp L1PA4 with antisense transcription initiated in its promoter. Transcription is split by strand (blue = forward; red = reverse). E) Comparison of the pseudo-bulk cluster expression of young L1 subfamilies among the different cell types (AP = apical progenitors; BP = basal progenitors; CR = Cajal Retzius; EBN = early-born neurons; IN = interneurons; M = microglia). F) Cluster expression of young L1 subfamilies (quantified per sample), grouped per cell type. G) L1 expression of cycling vs non-cycling cells from each cluster, grouped per cell type (p-value as per paired Wilcoxon test).

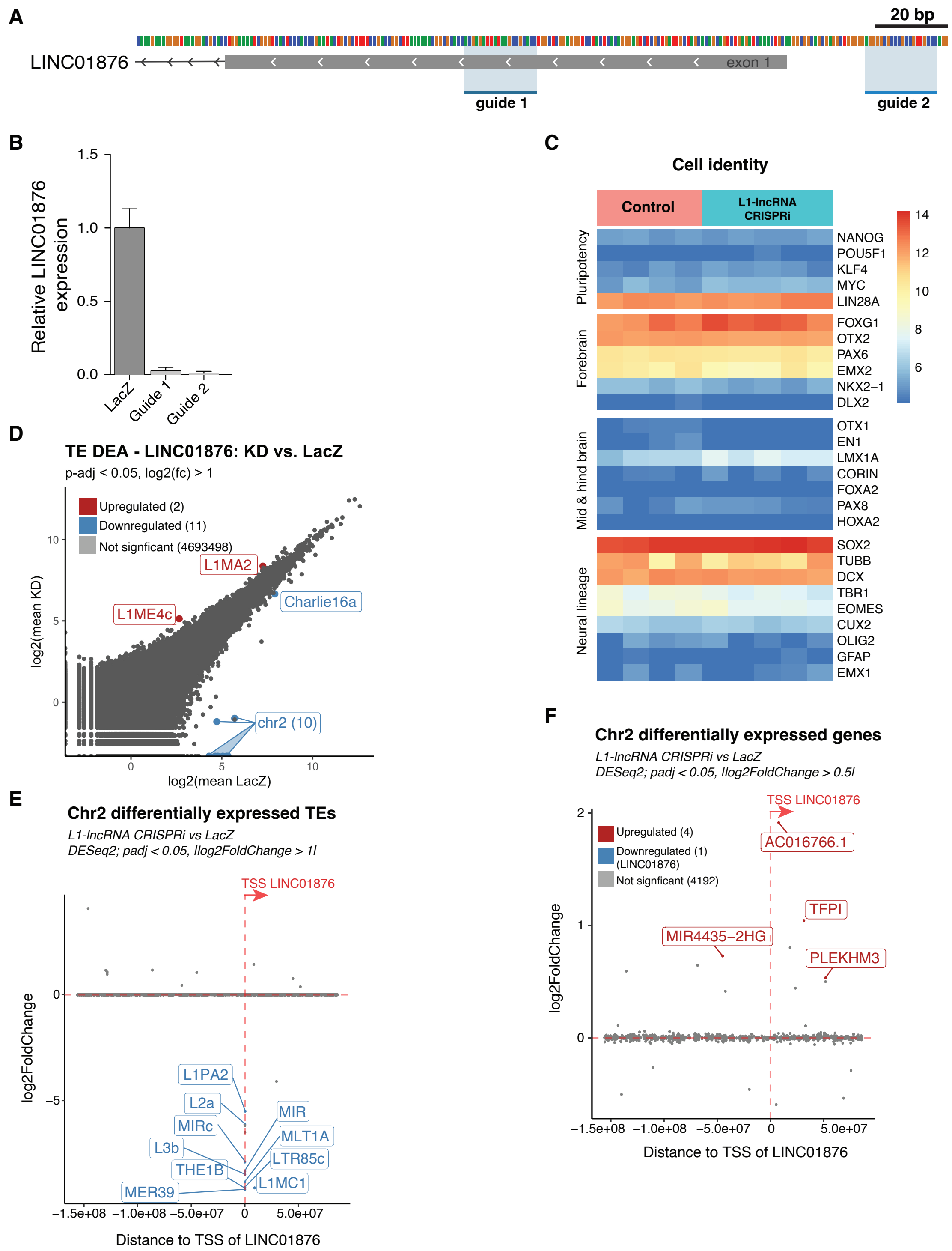

Figure S4 L1-lncRNA CRISPRi guide design and validation. A) Location of the CRISPRi guides targeting LINC01876. B) qPCR of L1-lncRNA in control (LacZ) and L1-lncRNA CRISPRi guide 1 and 2. C) Selected gene markers to represent cell identity in controls and L1-lncRNA fbNPCs. D) Mean plot showing results of differential expression analysis of individual TEs (DESeq2). Significantly upregulated elements (padj < 0.05; log2FoldChange >1) highlighted in red; significantly downregulated elements (padj < 0.05; log2FoldChange < -1) highlighted in blue. Labels showing TE subfamily or if the TE is located in chr2. E) Manhattan plot of chr2 showing log2FoldChange of TEs. Differentially expressed TEs nearby L1-lncRNA (start site at x = 0) are highlighted in red or blue (up and downregulated, respectively) (DESeq2, |log2FoldChange > 1|; padj < 0.05). F) Manhattan plot of chr2 showing log2FoldChange of genes. Differentially expressed genes nearby L1-lncRNA (start site at x = 0) are highlighted in red and blue (up and downregulated, respectively) (DESeq2, log2FoldChange > 0.5; padj < 0.05).

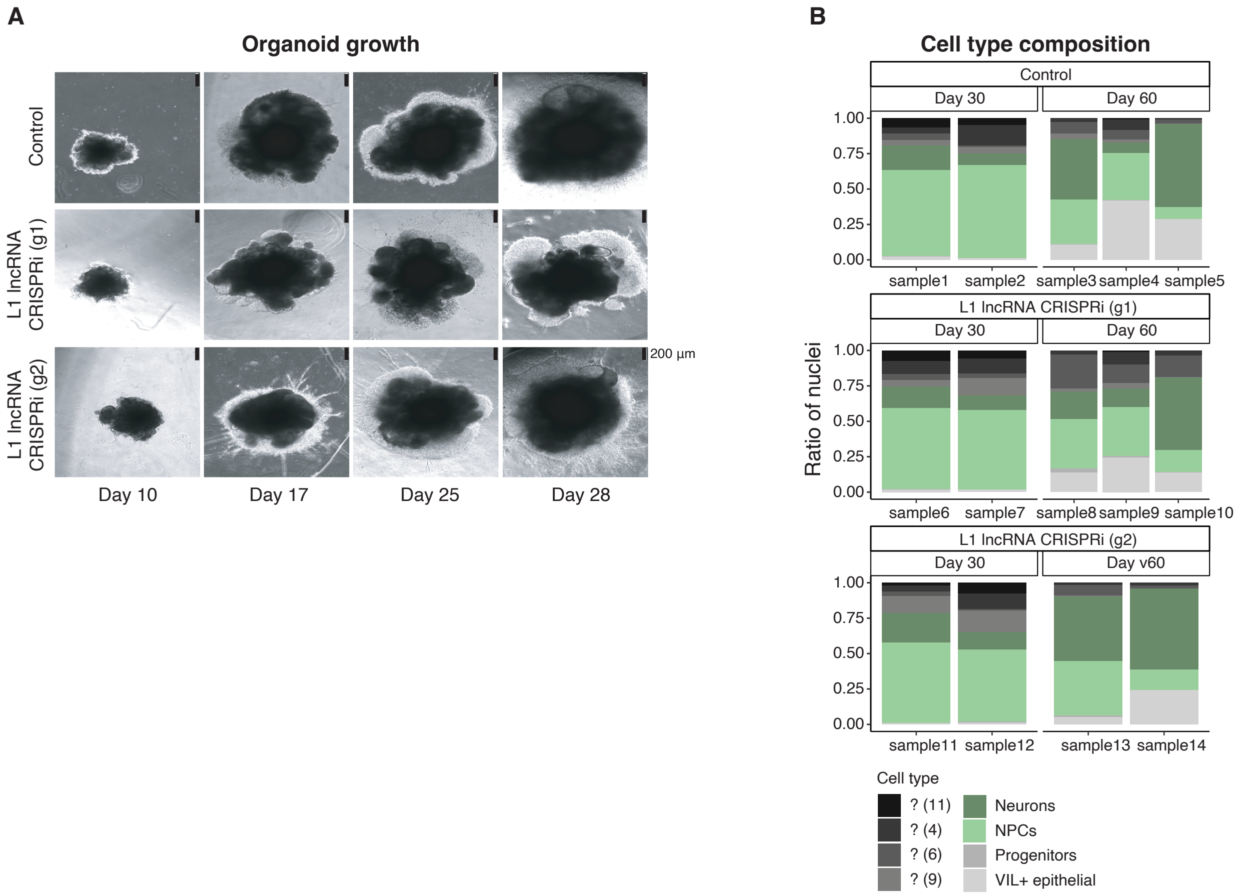

Figure S5 L1-lncRNA CRISPRi organoids size and composition. A) Brightfield imaging showing organoids sizes at day 10, 17, 25 and 28 (scale 200 µm, black bar) in control (LacZ) and L1-lncRNA CRISPRi (g1 and g2). B) Cell type composition in control (LacZ) and L1-lncRNA CRISPRi (g1 and g2) cerebral organoids. Neural-like cell types colored in green.

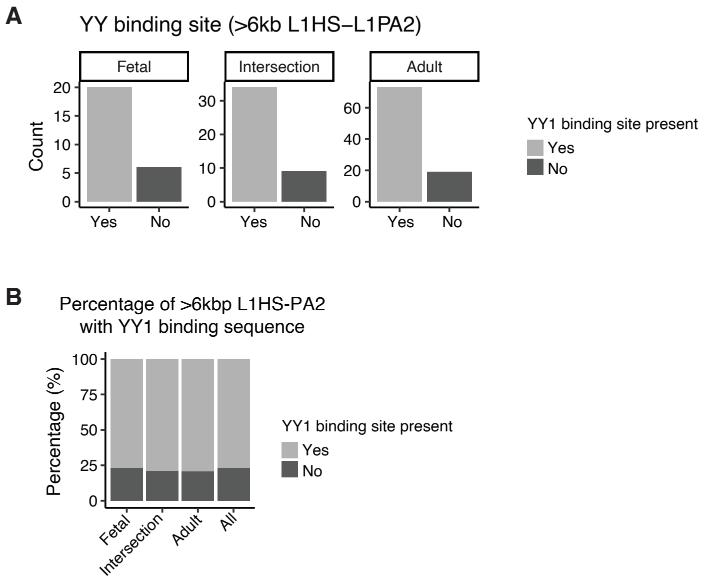

Figure S6 YY1-binding young L1s are expressed in the human brain. A) Number of expressed L1HS-L1PA2 (>6kbp) with YY1 binding sequence present (see methods) in fetal, adult, or those expressed in both datasets (intersection). B) Percentage of >6kbp L1HS-L1PA2 with YY1 binding sequence among those expressed in fetal samples, adult samples, both datasets (intersection), and all annotated in hg38 (see methods).

A)

Sample	Age	Sex	Region	Cause of death	PMI (hours)	Sequencing technology
CTL_501F	87	M	Frontal lobe	Acute myocardial infarction	48	Bulk RNAseq (short read, Illumina), snRNAseq (10x)
CTL_501T	87	M	Temporal lobe	Acute myocardial infarction	48	Bulk RNAseq (short read, Illumina), snRNAseq (10x), CUT&RUN (Illumina)
CTL_502T	75	M	Temporal lobe	Acute myocardial infarction	50	Bulk RNAseq (short read, Illumina), snRNAseq (10x)
CTL_502F	75	M	Frontal lobe	Acute myocardial infarction	50	Bulk RNAseq (short read, Illumina), CUT&RUN (Illumina)
CTL_529F	69	M	Frontal lobe	Disseminated malignant tumor and cicrulatory collapse (cardiac insufficiency)	95	Bulk RNAseq (short read, Illumina), snRNAseq (10x)
CTL_529T	69	M	Temporal lobe	Disseminated malignant tumor and cicrulatory collapse (cardiac insufficiency)	95	Bulk RNAseq (short read, Illumina), snRNAseq (10x), CUT&RUN (Illumina)
pt_088_003 or ASAP16-Ctl-PT231	84	M	Prefrontal cortex	Cardiac arrest secondary myocardial infarction. Ischaemic heart disease	73	PacBio Isoseq, snRNAseq (10x 5')
ASAP15-Ctl-NP16-21	83	M	Prefrontal cortex	N/A	N/A	snRNAseq (10x 5')
ASAP13_Ctl_NP16-161	72	M	Prefrontal cortex	N/A	N/A	snRNAseq (10x 5')

B)

Sample	Age	Region	Sequencing technology
DA817	7 weeks	Forebrain	CUT&RUN (Illumina)
DA103	8 weeks	Forebrain	Bulk RNAseq (short read, Illumina), snRNAseq (10x)
DA094	8 weeks	Forebrain	Bulk RNAseq (short read, Illumina), snRNAseq (10x)
DA140	9 weeks	Forebrain	Bulk RNAseq (short read, Illumina), snRNAseq (10x)
DA822	10 weeks	Forebrain	CUT&RUN (Illumina)
HuEmCtx_40mm_190611	10.5 weeks	Forebrain	Bulk RNAseq (short read, Illumina), snRNAseq (10x)
HuEmCtx_40mm_190611 (replicate)	10.5 weeks	Forebrain	Bulk RNAseq (short read, Illumina), snRNAseq (10x)
HuEmCtx_40mm_201029	10.5 weeks	Forebrain	Bulk RNAseq (short read, Illumina), snRNAseq (10x)

Supplemental Table 1 Demographics of adult and fetal samples including the brain region of sample collection and sequencing approach *A) adult cortex samples. PMI = Post-mortem interval. B) fetal forebrain samples*

A)

Day	Mean size (µm) ± SEM			Adjusted P-value	
	LacZ	Guide 1	Guide 2	LacZ vs Guide 1	LacZ vs Guide 2
10	470.24 ± 11.48	462.62 ± 9.97	464.94 ± 10.86	0.8913	0.9454
15	788.70 ± 13.60	809.50 ± 11.35	797.17 ± 12.29	0.4476	0.8681
17	952.24 ± 11.46	856.12 ± 14.02	867.95 ± 12.37	<0.0001	<0.0001
19	1018.77 ± 17.51	944.69 ± 13.46	918.06 ± 14.7	0.0003	<0.0001
21	1084.87 ± 12.20	1014.29 ± 16.45	998.09 ± 14.34	0.0005	<0.0001
23	1159.81 ± 15.01	1062.40 ± 13.55	1066.41 ± 19.16	<0.0001	<0.0001
25	1172.91 ± 22.28	1071.41 ± 22.99	1113.49 ± 13.35	<0.0001	0.0040
28	1256.89 ± 20.36	1150.60 ± 20.21	1152.38 ± 21.19	<0.0001	<0.0001
30	1273.00 ± 17.81	1190.78 ± 18.11	1186.29 ± 19.29	<0.0001	<0.0001

B)

Day	Mean size (µm) ± SEM		N		Adjusted P-value
	LacZ	LINC01876-CRISPi	LacZ	LINC01876-CRISPi	
10	470.24 ± 11.48	463.78 ± 7.31	30	60	>0.9999
15	788.70 ± 13.60	803.33 ± 8.33	30	60	0.9994
17	952.24 ± 11.46	862.04 ± 9.30	30	60	0.0010
19	1018.77 ± 17.51	931.37 ± 10.03	30	60	0.0016
21	1084.87 ± 12.20	1006.19 ± 10.87	30	60	0.0065
23	1159.81 ± 15.01	1064.40 ± 11.63	30	60	0.0004
25	1172.91 ± 22.28	1092.45 ± 13.46	30	60	0.0049
28	1256.89 ± 20.36	1151.49 ± 14.52	30	60	<0.0001
30	1273.00 ± 17.81	1188.53 ± 13.12	30	60	0.0026

Supplemental Table 2 Statistical analysis of organoid growth. *The size of 10 organoids was measured at each time point in three independent replicates of the experiment, for a total of 30 organoids per time point, per condition. A) Per CRISPRi guide RNA: Statistical analysis was performed using Two-way ANOVA and a Dunnett correction for multiple comparisons. B) Pooled CRISPRi guide RNAs: Statistical analysis was performed using Mixed-effects analysis and a Sidak correction for multiple comparisons.*

A)

transcript_id	expression (median of ratios)	gene_name	TE_chr	TE_start	TE_end	TE strand	TE_id
ENST00000255380.8	1527.673466	CHRM3	chr1	239623499	239629523	-	L1HS_1216
ENST00000307378.10	54.13908097	SLCO1A2	chr12	21395010	21401039	+	L1PA2_55013
ENST00000340333.7	140.3337054	ALG1L	chr3	125936639	125942696	+	L1PA2_52565
ENST00000340333.7	140.3337054	ALG1L	chr3	125936639	125942696	+	L1PA2_52565
ENST00000359720.3	24.09723964	MGST1	chr12	16377146	16383292	-	L1PA4_74761
ENST00000370904.6	138.2032631	IGSF1	chrX	131578681	131584735	+	L1PA2_53827
ENST00000451742.5	31.84167299	KCNMB2-AS1	chr3	178859949	178865979	+	L1PA2_52583
ENST00000471987.1	56.24307057	CD46	chr1	207770404	207776489	-	L1PA6_109103
ENST00000482821.5	246.7371293	MRPS5	chr2	95092744	95098852	+	L1PA6_97311
ENST00000525504.5	131.5739232	CCDC90B	chr11	83266135	83272255	+	L1PA6_94344
ENST00000552624.5	429.5793945	SYT1	chr12	79039622	79045645	-	L1PA2_54103
ENST00000552744.5	114.9020407	SYT1	chr12	79039622	79045645	-	L1PA2_54103
ENST00000560054.6	48.35696725	RP11-499F3.2	chr15	81870668	81876699	+	L1PA2_50395
ENST00000588311.5	120.7950971	ZNF567	chr19	36697924	36704066	-	L1PA3_67830
ENST00000591567.1	75.07247056	RP11-120M18.2	chr17	68408124	68414142	-	L1PA3_54232
ENST00000592030.1	34.69973891	RP11-120M18.2	chr17	68408124	68414142	-	L1PA3_54232
ENST00000639821.1	36.88242567	ADGRV1	chr5	90923631	90929658	-	L1PA2_56284
ENST00000649519.1	23.59199316	FANCC	chr9	95254281	95260702	+	L1PA7_109805
ENST00000655360.1	77.54124278	CTD-2297M2.1	chr5	88052331	88058345	+	L1PA2_63036
ENST00000656175.1	39.25527246	AC009312.1	chr2	117803781	117809809	+	L1PA3_54280
ENST00000659876.1	42.84268982	AC069277.2	chr3	6484912	6490929	-	L1PA2_56147
ENST00000661045.1	25.27304418	LINC01748	chr1	60640013	60646037	+	L1PA2_52219
ENST00000662846.1	21.57055305	AC069277.2	chr3	6484912	6490929	-	L1PA2_56147
ENST00000665712.1	121.9865528	RP11-527D7.1	chr1	241424986	241431394	-	L1PA7_11540
MSTRG.13606.14	153045.6095		chr12	79039622	79045645	-	L1PA2_54103
MSTRG.13606.15	176.6487261		chr12	79039622	79045645	-	L1PA2_54103
MSTRG.17071.4	3469.698427		chr14	52590835	52596957	-	L1PA6_103786
MSTRG.32316.30	21.34145396		chr2	71411475	71417501	-	L1HS_1101
MSTRG.43045.1	1679.484134		chr4	165849	171989	-	L1PA4_90148
MSTRG.54890.1	1029.608783		chr7	128292012	128298140	+	L1PA3_65221
MSTRG.56072.11	3160.865438		chr8	15555982	15562028	-	L1PA2_54695
MSTRG.62073.1	3851.141577		chrX	76978612	76984765	+	L1PA3_83092

B)

transcript_id	expression (median of ratios)	gene_name	TE_chr	TE_start	TE_end	TE strand	TE_id
ENST00000255380.8	81.476946	CHRM3	chr1	2.4E+08	239629523	-	L1HS_1216
ENST00000307378.10	56.54165	SLCO1A2	chr12	21395010	21401039	+	L1PA2_55013
ENST00000340333.7	33.130089	ALG1L	chr3	1.26E+08	125942696	+	L1PA2_52565
ENST00000340333.7	33.130089	ALG1L	chr3	1.26E+08	125942696	+	L1PA2_52565
ENST00000357564.4	24.259704	POC5	chr5	75695699	75701728	+	L1PA3_57248
ENST00000370904.6	115.41432	IGSF1	chrX	1.32E+08	131584735	+	L1PA2_53827
ENST00000428651.2	44.369475	LINC01876	chr2	1.56E+08	156260532	+	L1PA2_52499
ENST00000428651.2	44.369475	LINC01876	chr2	1.56E+08	156260532	+	L1PA2_52499
ENST00000457110.1	22.77516	AC009312.1	chr2	1.18E+08	117809809	+	L1PA3_54280
ENST00000471987.1	205.794	CD46	chr1	2.08E+08	207776489	-	L1PA6_109103
ENST00000482821.5	821.92121	MRPS5	chr2	95092744	95098852	+	L1PA6_97311
ENST00000507400.1	38.678392	TUSC3	chr8	15555982	15562028	-	L1PA2_54695
ENST00000525504.5	198.75191	CCDC90B	chr11	83266135	83272255	+	L1PA6_94344
ENST00000559573.3	44.609517	FAM227B	chr15	49395984	49402014	+	L1PA2_52408
ENST00000560054.6	28.211006	RP11-499F3.2	chr15	81870668	81876699	+	L1PA2_50395
ENST00000588874.5	410.2802	BCAS3	chr17	60881441	60887474	-	L1PA2_4326
ENST00000591567.1	27.190991	RP11-120M18.2	chr17	68408124	68414142	-	L1PA3_54232
ENST00000592030.1	31.800684	RP11-120M18.2	chr17	68408124	68414142	-	L1PA3_54232
ENST00000611052.4	26.657673	RP11-726G1.2	chr12	9627299	9633386	-	L1PA5_100889
ENST00000626966.2	595.71002	PLCB1	chr20	8595102	8601127	-	L1PA2_5409
ENST00000635799.1	26.805454	LINC01876	chr2	1.56E+08	156260532	+	L1PA2_52499
ENST00000635799.1	26.805454	LINC01876	chr2	1.56E+08	156260532	+	L1PA2_52499
ENST00000639821.1	48.095288	ADGRV1	chr5	90923631	90929658	-	L1PA2_56284
ENST00000653487.1	42.222436	CTC-523E23.11	chr19	34860178	34866213	+	L1PA4_53293
ENST00000655360.1	48.726987	CTD-2297M2.1	chr5	88052331	88058345	+	L1PA2_63036
ENST00000655389.1	99.566579	CTC-523E23.11	chr19	34860178	34866213	+	L1PA4_53293
ENST00000655441.1	55.32148	RP11-175O15.1	chr2	1.85E+08	185425462	-	L1PA2_51796
ENST00000655441.1	55.32148	RP11-175O15.1	chr2	1.85E+08	185425462	-	L1PA2_51796
ENST00000656175.1	366.53158	AC009312.1	chr2	1.18E+08	117809809	+	L1PA3_54280
ENST00000657334.1	20.078974	LINC01876	chr2	1.56E+08	156260532	+	L1PA2_52499
ENST00000657334.1	20.078974	LINC01876	chr2	1.56E+08	156260532	+	L1PA2_52499
ENST00000659237.1	22.170288	CASC15	chr6	22174841	22180874	-	L1PA2_51341
ENST00000659502.1	26.844311	LINC01876	chr2	1.56E+08	156260532	+	L1PA2_52499
ENST00000659502.1	26.844311	LINC01876	chr2	1.56E+08	156260532	+	L1PA2_52499
ENST00000659876.1	73.159983	AC069277.2	chr3	6484912	6490929	-	L1PA2_56147
ENST00000661045.1	88.734273	LINC01748	chr1	60640013	60646037	+	L1PA2_52219
ENST00000661513.1	59.16715	LINC01876	chr2	1.56E+08	156260532	+	L1PA2_52499
ENST00000661513.1	59.16715	LINC01876	chr2	1.56E+08	156260532	+	L1PA2_52499
ENST00000662065.1	72.595773	LINC01748	chr1	60640013	60646037	+	L1PA2_52219
ENST00000669870.1	32.869951	LINC02893	chr9	87041883	87047900	+	L1PA3_56510
MSTRG.13606.15	113.34061		chr12	79039622	79045645	-	L1PA2_54103
MSTRG.17071.4	2320.2294		chr14	52590835	52596957	-	L1PA6_103786
MSTRG.29341.3	2258.4253		chr19	34860178	34866213	+	L1PA4_53293
MSTRG.32316.30	818.00695		chr2	71411475	71417501	-	L1HS_1101
MSTRG.42852.5	437.63463		chr3	1.95E+08	195093677	+	L1PA2_52590
MSTRG.43045.1	3692.0677		chr4	165849	171989	-	L1PA4_90148

MSTRG.54049.8	1351.0883		chr7	87039757	87045768	+	L1PA2_54667
MSTRG.54049.9	1210.482		chr7	87039757	87045768	+	L1PA2_54667
MSTRG.54890.1	634.92769		chr7	1.28E+08	128298140	+	L1PA3_65221
MSTRG.56072.11	6945.1786		chr8	15555982	15562028	-	L1PA2_54695
MSTRG.62020.5	3077.699		chrX	74474479	74480631	-	L1PA4_87627
MSTRG.62073.1	284.97116		chrX	76978612	76984765	+	L1PA3_83092

Supplemental Table 3 L1-chimera transcripts. *Transcripts ids and gene names as annotated in gencode v38 (or* de novo*), transcript expression level, and coordinates of the L1 residing in the transcript's promoter site. A) Transcripts expressed in adult samples B) Transcripts expressed in fetal samples.*

REFERENCES AND NOTES

1. R. S. Hill, C. A. Walsh, Molecular insights into human brain evolution. *Nature* **437**, 64–67 (2005).

2. J. H. Lui, D. V. Hansen, A. R. Kriegstein, Development and evolution of the human neocortex. *Cell* **146**, 18–36 (2011).

3. P. Rakic, Evolution of the neocortex: A perspective from developmental biology. *Nat. Rev. Neurosci.* **10**, 724–735 (2009).

4. A. M. M. Sousa, K. A. Meyer, G. Santpere, F. O. Gulden, N. Sestan, Evolution of the human nervous system function, structure, and development. *Cell* **170**, 226–247 (2017).

5. Chimpanzee Sequencing and Analysis Consortium, Initial sequence of the chimpanzee genome and comparison with the human genome. *Nature* **437**, 69–87 (2005).

6. Z. N. Kronenberg, I. T. Fiddes, D. Gordon, S. Murali, S. Cantsilieris, O. S. Meyerson, J. G. Underwood, B. J. Nelson, M. J. P. Chaisson, M. L. Dougherty, K. M. Munson, A. R. Hastie, M. Diekhans, F. Hormozdiari, N. Lorusso, K. Hoekzema, R. Qiu, K. Clark, A. Raja, A. E. Welch, M. Sorensen, C. Baker, R. S. Fulton, J. Armstrong, T. A. Graves-Lindsay, A. M. Denli, E. R. Hoppe, P. Hsieh, C. M. Hill, A. W. C. Pang, J. Lee, E. T. Lam, S. K. Dutcher, F. H. Gage, W. C. Warren, J. Shendure, D. Haussler, V. A. Schneider, H. Cao, M. Ventura, R. K. Wilson, B. Paten, A. Pollen, E. E. Eichler, High-resolution comparative analysis of great ape genomes. *Science* **360**, eaar6343 (2018).

7. S. J. Hoyt, J. M. Storer, G. A. Hartley, P. G. Grady, A. Gershman, L. G. de Lima, C. Limouse, R. Halabian, L. Wojenski, M. Rodriguez, N. Altemose, A. Rhie, L. J. Core, J. L. Gerton, W. Makalowski, D. Olson, J. Rosen, A. F. A. Smit, A. F. Straight, M. R. Vollger, T. J. Wheeler, M. C. Schatz, E. E. Eichler, A. M. Phillippy, W. Timp, K. H. Miga, R. J. O'Neill, From telomere to telomere: The transcriptional and epigenetic state of human repeat elements. *Science* **376**, eabk3112 (2022).

8. R. Cordaux, M. A. Batzer, The impact of retrotransposons on human genome evolution. *Nat. Rev. Genet.* **10**, 691–703 (2009).

9. A. D. Ewing, H. H. Kazazian, Jr., High-throughput sequencing reveals extensive variation in human-specific L1 content in individual human genomes. *Genome Res.* **20**, 1262–1270 (2010).

10. M. E. Jonsson, R. Garza, P. A. Johansson, J. Jakobsson, Transposable elements: A common feature of neurodevelopmental and neurodegenerative disorders. *Trends Genet.* **36**, 610–623 (2020).

11. H. H. Kazazian, Jr., J. V. Moran, Mobile DNA in health and disease. *N. Engl. J. Med.* **377**, 361–370 (2017).

12. O. Deniz, J. M. Frost, M. R. Branco, Regulation of transposable elements by DNA modifications. *Nat. Rev. Genet.* **20**, 417–431 (2019).

13. J. L. Goodier, Restricting retrotransposons: A review. *Mob. DNA* **7**, 16 (2016).

14. M. E. Jönsson, P. Ludvik Brattas, C. Gustafsson, R. Petri, D. Yudovich, K. Pircs, S. Verschuere, S. Madsen, J. Hansson, J. Larsson, R. Mansson, A. Meissner, J. Jakobsson, Activation of neuronal genes via LINE-1 elements upon global DNA demethylation in human neural progenitors. *Nat. Commun.* **10**, 3182 (2019).

15. C. P. Walsh, J. R. Chaillet, T. H. Bestor, Transcription of IAP endogenous retroviruses is constrained by cytosine methylation. *Nat. Genet.* **20**, 116–117 (1998).

16. E. B. Chuong, N. C. Elde, C. Feschotte, Regulatory activities of transposable elements: From conflicts to benefits. *Nat. Rev. Genet.* **18**, 71–86 (2017).

17. A. Kapusta, Z. Kronenberg, V. J. Lynch, X. Zhuo, L. Ramsay, G. Bourque, M. Yandell, C. Feschotte, Transposable elements are major contributors to the origin, diversification, and regulation of vertebrate long noncoding RNAs. *PLOS Genet.* **9**, e1003470 (2013).

18. J. L. Rinn, H. Y. Chang, Long noncoding RNAs: Molecular modalities to organismal functions. *Annu. Rev. Biochem.* **89**, 283–308 (2020).

19. C. R. Beck, J. L. Garcia-Perez, R. M. Badge, J. V. Moran, LINE-1 elements in structural variation and disease. *Annu. Rev. Genomics Hum. Genet.* **12**, 187–215 (2011).

20. E. S. Lander, L. M. Linton, B. Birren, C. Nusbaum, M. C. Zody, J. Baldwin, K. Devon, K. Dewar, M. Doyle, W. FitzHugh, R. Funke, D. Gage, K. Harris, A. Heaford, J. Howland, L. Kann, J. Lehoczky, R. LeVine, P. McEwan, K. McKernan, J. Meldrim, J. P. Mesirov, C. Miranda, W. Morris, J. Naylor, C. Raymond, M. Rosetti, R. Santos, A. Sheridan, C. Sougnez, Y. Stange-Thomann, N. Stojanovic, A. Subramanian, D. Wyman, J. Rogers, J. Sulston, R. Ainscough, S. Beck, D. Bentley, J. Burton, C. Clee, N. Carter, A. Coulson, R. Deadman, P. Deloukas, A. Dunham, I. Dunham, R. Durbin, L. French, D. Grafham, S. Gregory, T. Hubbard, S. Humphray, A. Hunt, M. Jones, C. Lloyd, A. McMurray, L. Matthews, S. Mercer, S. Milne, J. C. Mullikin, A. Mungall, R. Plumb, M. Ross, R. Shownkeen, S. Sims, R. H. Waterston, R. K. Wilson, L. W. Hillier, J. D. McPherson, M. A. Marra, E. R. Mardis, L. A. Fulton, A. T. Chinwalla, K. H. Pepin, W. R. Gish, S. L. Chissoe, M. C. Wendl, K. D. Delehaunty, T. L. Miner, A. Delehaunty, J. B. Kramer, L. L. Cook, R. S. Fulton, D. L. Johnson, P. J. Minx, S. W. Clifton, T. Hawkins, E. Branscomb, P. Predki, P. Richardson, S. Wenning, T. Slezak, N. Doggett, J. F. Cheng, A. Olsen, S. Lucas, C. Elkin, E. Uberbacher, M. Frazier, R. A. Gibbs, D. M. Muzny, S. E. Scherer, J. B. Bouck, E. J. Sodergren, K. C. Worley, C. M. Rives, J. H. Gorrell, M. L. Metzker, S. L. Naylor, R. S. Kucherlapati, D. L. Nelson, G. M. Weinstock, Y. Sakaki, A. Fujiyama, M. Hattori, T. Yada, A. Toyoda, T. Itoh, C. Kawagoe, H. Watanabe, Y. Totoki, T. Taylor, J. Weissenbach, R. Heilig, W. Saurin, F. Artiguenave, P. Brottier, T. Bruls, E. Pelletier, C. Robert, P. Wincker, D. R. Smith, L. Doucette-Stamm, M. Rubenfield, K. Weinstock, H. M. Lee, J. Dubois, A. Rosenthal, M. Platzer, G. Nyakatura, S. Taudien, A. Rump, H. Yang, J. Yu, J. Wang, G. Huang, J. Gu, L. Hood, L. Rowen, A. Madan, S. Qin, R. W. Davis, N. A. Federspiel, A. P. Abola, M. J. Proctor, R. M. Myers, J. Schmutz, M. Dickson, J. Grimwood, D. R. Cox, M. V. Olson, R. Kaul, C. Raymond, N. Shimizu, K. Kawasaki, S. Minoshima, G. A. Evans, M. Athanasiou, R. Schultz, B. A. Roe, F. Chen, H. Pan, J. Ramser, H. Lehrach, R. Reinhardt, W. R. McCombie, M. de la Bastide, N. Dedhia, H. Blocker, K. Hornischer, G. Nordsiek, R. Agarwala, L. Aravind, J. A. Bailey, A. Bateman, S. Batzoglou, E. Birney, P. Bork, D. G. Brown, C. B. Burge, L. Cerutti, H. C. Chen, D. Church, M. Clamp, R. R. Copley, T. Doerks, S. R. Eddy, E. E. Eichler, T. S. Furey, J. Galagan, J. G. Gilbert, C. Harmon, Y. Hayashizaki, D. Haussler, H. Hermjakob, K. Hokamp, W. Jang, L. S. Johnson, T. A. Jones, S. Kasif, A. Kaspryzk, S. Kennedy, W. J. Kent, P. Kitts, E. V. Koonin, I. Korf, D. Kulp, D. Lancet, T. M. Lowe, A. McLysaght, T. Mikkelsen, J. V. Moran, N. Mulder, V. J. Pollara, C. P. Ponting, G. Schuler, J. Schultz, G. Slater, A. F. Smit, E. Stupka, J. Szustakowki, D. Thierry-Mieg, J. Thierry-Mieg, L. Wagner, J. Wallis, R. Wheeler, A. Williams, Y. I. Wolf, K. H. Wolfe, S. P. Yang,

R. F. Yeh, F. Collins, M. S. Guyer, J. Peterson, A. Felsenfeld, K. A. Wetterstrand, A. Patrinos, M. J. Morgan, P. de Jong, J. J. Catanese, K. Osoegawa, H. Shizuya, S. Choi, Y. J. Chen, J. Szustakowki; International Human Genome Sequencing Consortium, Initial sequencing and analysis of the human genome. *Nature* **409**, 860–921 (2001).

21. H. Khan, A. Smit, S. Boissinot, Molecular evolution and tempo of amplification of human LINE-1 retrotransposons since the origin of primates. *Genome Res.* **16**, 78–87 (2006).

22. Q. Feng, J. V. Moran, H. H. Kazazian, Jr, J. D. Boeke, Human L1 retrotransposon encodes a conserved endonuclease required for retrotransposition. *Cell* **87**, 905–916 (1996).

23. S. L. Mathias, A. F. Scott, H. H. Kazazian, Jr, J. D. Boeke, A. Gabriel, Reverse transcriptase encoded by a human transposable element. *Science* **254**, 1808–1810 (1991).

24. G. D. Swergold, Identification, characterization, and cell specificity of a human LINE-1 promoter. *Mol. Cell. Biol.* **10**, 6718–6729 (1990).

25. A. M. Denli, I. Narvaiza, B. E. Kerman, M. Pena, C. Benner, M. C. Marchetto, J. K. Diedrich, A. Aslanian, J. Ma, J. J. Moresco, L. Moore, T. Hunter, A. Saghatelian, F. H. Gage, Primate-specific ORF0 contributes to retrotransposon-mediated diversity. *Cell* **163**, 583–593 (2015).

26. M. Speek, Antisense promoter of human L1 retrotransposon drives transcription of adjacent cellular genes. *Mol. Cell. Biol.* **21**, 1973–1985 (2001).

27. N. G. Coufal, J. L. Garcia-Perez, G. E. Peng, G. W. Yeo, Y. Mu, M. T. Lovci, M. Morell, K. S. O'Shea, J. V. Moran, F. H. Gage, L1 retrotransposition in human neural progenitor cells. *Nature* **460**, 1127–1131 (2009).

28. J. A. Erwin, A. C. Paquola, T. Singer, I. Gallina, M. Novotny, C. Quayle, T. A. Bedrosian, F. I. Alves, C. R. Butcher, J. R. Herdy, A. Sarkar, R. S. Lasken, A. R. Muotri, F. H. Gage, L1-associated genomic regions are deleted in somatic cells of the healthy human brain. *Nat. Neurosci.* **19**, 1583–1591 (2016).

29. G. D. Evrony, X. Cai, E. Lee, L. B. Hills, P. C. Elhosary, H. S. Lehmann, J. J. Parker, K. D. Atabay, E. C. Gilmore, A. Poduri, P. J. Park, C. A. Walsh, Single-neuron sequencing analysis of L1 retrotransposition and somatic mutation in the human brain. *Cell* **151**, 483–496 (2012).

30. G. D. Evrony, E. Lee, B. K. Mehta, Y. Benjamini, R. M. Johnson, X. Cai, L. Yang, P. Haseley, H. S. Lehmann, P. J. Park, C. A. Walsh, Cell lineage analysis in human brain using endogenous retroelements. *Neuron* **85**, 49–59 (2015).

31. A. R. Muotri, V. T. Chu, M. C. N. Marchetto, W. Deng, J. V. Moran, F. H. Gage, Somatic mosaicism in neuronal precursor cells mediated by L1 retrotransposition. *Nature* **435**, 903–910 (2005).

32. F. J. Sanchez-Luque, M.-J. H. C. Kempen, P. Gerdes, D. B. Vargas-Landin, S. R. Richardson, R.-L. Troskie, J. S. Jesuadian, S. W. Cheetham, P. E. Carreira, C. Salvador-Palomeque, M. Garcia-Cañadas, M. Muñoz-Lopez, L. Sanchez, M. Lundberg, A. Macia, S. R. Heras, P. M. Brennan, R. Lister, J. L. Garcia-Perez, A. D. Ewing, G. J. Faulkner, LINE-1 evasion of epigenetic repression in humans. *Mol. Cell* **75**, 590–604.e12 (2019).

33. K. R. Upton, D. J. Gerhardt, J. S. Jesuadian, S. R. Richardson, F. J. Sanchez-Luque, G. O. Bodea, A. D. Ewing, C. Salvador-Palomeque, M. S. van der Knaap, P. M. Brennan, A. Vanderver, G. J. Faulkner, Ubiquitous L1 mosaicism in hippocampal neurons. *Cell* **161**, 228–239 (2015).

34. S. Lanciano, G. Cristofari, Measuring and interpreting transposable element expression. *Nat. Rev. Genet.* **21**, 721–736 (2020).

35. Y. Jin, O. H. Tam, E. Paniagua, M. Hammell, *TEtranscripts*: A package for including transposable elements in differential expression analysis of RNA-seq datasets. *Bioinformatics* **31**, 3593–3599 (2015).

36. M. E. Jonsson, R. Garza, Y. Sharma, R. Petri, E. Sodersten, J. G. Johansson, P. A. Johansson, D. A. Atacho, K. Pircs, S. Madsen, D. Yudovich, R. Ramakrishnan, J. Holmberg, J. Larsson, P. Jern, J. Jakobsson, Activation of endogenous retroviruses during brain development causes an inflammatory response. *EMBO J.* **40**, e106423 (2021).

37. V. P. Belancio, M. Whelton, P. Deininger, Requirements for polyadenylation at the 3′ end of LINE-1 elements. *Gene* **390**, 98–107 (2007).

38. E. M. Ostertag, H. H. Kazazian, Jr, Twin priming: A proposed mechanism for the creation of inversions in L1 retrotransposition. *Genome Res.* **11**, 2059–2065 (2001).

39. E. K. Gustavsson, S. Sethi, Y. Gao, J. W. Brenton, S. García-Ruiz, D. Zhang, R. Garza, R. H. Reynolds, J. R. Evans, Z. Chen, M. Grant-Peters, H. Macpherson, K. Montgomery, R. Dore, A. I. Wernick, C. Arber, S. Wray, S. Gandhi, J. Esselborn, C. Blauwendraat, C. H. Douse, A. Adami, D. A. M. Atacho, A. Kouli, A. Quaegebeur, R. A. Barker, E. Englund, F. Platt, J. Jakobsson, N. W. Wood, H. Houlden, H. Saini, C. F. Bento, J. Hardy, M. Ryten, The annotation and function of the Parkinson's and Gaucher disease-linked gene GBA1 has been concealed by its protein-coding pseudogene GBAP1 (Cold Spring Harbor Laboratory, 2022).

40. J. Feusier, W. S. Watkins, J. Thomas, A. Farrell, D. J. Witherspoon, L. Baird, H. Ha, J. Xing, L. B. Jorde, Pedigree-based estimation of human mobile element retrotransposition rates. *Genome Res.* **29**, 1567–1577 (2019).

41. P. J. Skene, S. Henikoff, An efficient targeted nuclease strategy for high-resolution mapping of DNA binding sites. *eLife* **6**, e21856 (2017).

42. B. Brouha, J. Schustak, R. M. Badge, S. Lutz-Prigge, A. H. Farley, J. V. Moran, H. H. Kazazian, Jr., Hot L1s account for the bulk of retrotransposition in the human population. *Proc. Natl. Acad. Sci. U.S.A.* **100**, 5280–5285 (2003).

43. G. La Manno, R. Soldatov, A. Zeisel, E. Braun, H. Hochgerner, V. Petukhov, K. Lidschreiber, M. E. Kastriti, P. Lonnerberg, A. Furlan, J. Fan, L. E. Borm, Z. Liu, D. van Bruggen, J. Guo, X. He, R. Barker, E. Sundstrom, G. Castelo-Branco, P. Cramer, I. Adameyko, S. Linnarsson, P. V. Kharchenko, RNA velocity of single cells. *Nature* **560**, 494–498 (2018).

44. M. Astick, P. Vanderhaeghen, From human pluripotent stem cells to cortical circuits. *Curr. Top. Dev. Biol.* **129**, 67–98 (2018).

45. C. Philippe, D. B. Vargas-Landin, A. J. Doucet, D. van Essen, J. Vera-Otarola, M. Kuciak, A. Corbin, P. Nigumann, G. Cristofari, Activation of individual L1 retrotransposon instances is restricted to cell-type dependent permissive loci. *eLife* **5**, e13926 (2016).

46. P. A. Johansson, P. L. Brattas, C. H. Douse, P. Hsieh, A. Adami, J. Pontis, D. Grassi, R. Garza, E. Sozzi, R. Cataldo, M. E. Jonsson, D. A. M. Atacho, K. Pircs, F. Eren, Y. Sharma, J. Johansson, A. Fiorenzano, M. Parmar, M. Fex, D. Trono, E. E. Eichler, J. Jakobsson, A *cis*-acting structural variation at the ZNF558 locus controls a gene regulatory network in human brain development. *Cell Stem Cell* **29**, 52–69.e8 (2022).

47. S. B. Linker, I. Narvaiza, J. Y. Hsu, M. Wang, F. Qiu, A. P. D. Mendes, R. Oefner, K. Kottilil, A. Sharma, L. Randolph-Moore, E. Mejia, R. Santos, M. C. Marchetto, F. H. Gage, Human-specific regulation of neural maturation identified by cross-primate transcriptomics. *Curr. Biol.* **32**, 4797–4807.e5 (2022).

48. S. Kanton, M. J. Boyle, Z. He, M. Santel, A. Weigert, F. Sanchis-Calleja, P. Guijarro, L. Sidow, J. S. Fleck, D. Han, Z. Qian, M. Heide, W. B. Huttner, P. Khaitovich, S. Paabo, B. Treutlein, J. G. Camp, Organoid single-cell genomic atlas uncovers human-specific features of brain development. *Nature* **574**, 418–422 (2019).

49. Y. Mao, W. T. Harvey, D. Porubsky, K. M. Munson, K. Hoekzema, A. P. Lewis, P. A. Audano, A. Rozanski, X. Yang, S. Zhang, D. S. Gordon, X. Wei, G. A. Logsdon, M. Haukness, P. C. Dishuck, H. Jeong, R. Del Rosario, V. L. Bauer, W. T. Fattor, G. K. Wilkerson, Q. Lu, B. Paten, G. Feng, S. L. Sawyer, W. C. Warren, L. Carbone, E. E. Eichler, Structurally divergent and recurrently mutated regions of primate genomes (Cold Spring Harbor Laboratory, 2023).

50. S. Banfi, A. Servadio, M. Y. Chung, T. J. Kwiatkowski Jr, A. E. McCall, L. A. Duvick, Y. Shen, E. J. Roth, H. T. Orr, H. Y. Zoghbi, Identification and characterization of the gene causing type 1 spinocerebellar ataxia. *Nat. Genet.* **7**, 513–520 (1994).

51. J. M. Dewing, R. O. Carare, A. J. Lotery, J. A. Ratnayaka, The diverse roles of TIMP-3: Insights into degenerative diseases of the senescent retina and brain. *Cells* **9**, 39 (2019).

52. M. A. Lancaster, M. Renner, C. A. Martin, D. Wenzel, L. S. Bicknell, M. E. Hurles, T. Homfray, J. M. Penninger, A. P. Jackson, J. A. Knoblich, Cerebral organoids model human brain development and microcephaly. *Nature* **501**, 373–379 (2013).

53. V. P. Belancio, A. M. Roy-Engel, R. R. Pochampally, P. Deininger, Somatic expression of LINE-1 elements in human tissues. *Nucleic Acids Res.* **38**, 3909–3922 (2010).

54. G. J. Faulkner, Y. Kimura, C. O. Daub, S. Wani, C. Plessy, K. M. Irvine, K. Schroder, N. Cloonan, A. L. Steptoe, T. Lassmann, K. Waki, N. Hornig, T. Arakawa, H. Takahashi, J. Kawai, A. R. Forrest, H. Suzuki, Y. Hayashizaki, D. A. Hume, V. Orlando, S. M. Grimmond, P. Carninci, The regulated retrotransposon transcriptome of mammalian cells. *Nat. Genet.* **41**, 563–571 (2009).

55. S. Horvath, DNA methylation age of human tissues and cell types. *Genome Biol.* **14**, R115 (2013).

56. M. De Cecco, T. Ito, A. P. Petrashen, A. E. Elias, N. J. Skvir, S. W. Criscione, A. Caligiana, G. Brocculi, E. M. Adney, J. D. Boeke, O. Le, C. Beausejour, J. Ambati, K. Ambati, M. Simon, A. Seluanov, V. Gorbunova, P. E. Slagboom, S. L. Helfand, N. Neretti, J. M. Sedivy, L1 drives IFN in senescent cells and promotes age-associated inflammation. *Nature* **566**, 73–78 (2019).

57. M. Van Meter, M. Kashyap, S. Rezazadeh, A. J. Geneva, T. D. Morello, A. Seluanov, V. Gorbunova, SIRT6 represses LINE1 retrotransposons by ribosylating KAP1 but this repression fails with stress and age. *Nat. Commun.* **5**, 5011 (2014).

58. D. Ardeljan, J. P. Steranka, C. Liu, Z. Li, M. S. Taylor, L. M. Payer, M. Gorbounov, J. S. Sarnecki, V. Deshpande, R. H. Hruban, J. D. Boeke, D. Fenyo, P. H. Wu, A. Smogorzewska, A. J. Holland, K. H. Burns, Cell fitness screens reveal a conflict between LINE-1 retrotransposition and DNA replication. *Nat. Struct. Mol. Biol.* **27**, 168–178 (2020).

59. N. R. Wray, S. Ripke, M. Mattheisen, M. Trzaskowski, E. M. Byrne, A. Abdellaoui, M. J. Adams, E. Agerbo, T. M. Air, T. M. F. Andlauer, S.-A. Bacanu, M. Bækvad-Hansen, A. F. T. Beekman, T. B. Bigdeli, E. B. Binder, D. R. H. Blackwood, J. Bryois, H. N. Buttenschøn, J. Bybjerg-Grauholm, N. Cai, E. Castelao, J. H. Christensen, T.-K. Clarke, J. I. R. Coleman, L. Colodro-Conde, B. Couvy-Duchesne, N. Craddock, G. E. Crawford, C. A. Crowley, H. S. Dashti, G. Davies, I. J. Deary, F.

Degenhardt, E. M. Derks, N. Direk, C. V. Dolan, E. C. Dunn, T. C. Eley, N. Eriksson, V. Escott-Price, Farnush Hassan Farhadi Kiadeh, H. K. Finucane, A. J. Forstner, J. Frank, H. A. Gaspar, M. Gill, P. Giusti-Rodríguez, F. S. Goes, S. D. Gordon, J. Grove, L. S. Hall, E. Hannon, C. S. Hansen, T. F. Hansen, S. Herms, I. B. Hickie, P. Hoffmann, G. Homuth, C. Horn, J.-J. Hottenga, D. M. Hougaard, M. Hu, C. L. Hyde, M. Ising, R. Jansen, F. Jin, E. Jorgenson, J. A. Knowles, I. S. Kohane, J. Kraft, W. W. Kretzschmar, J. Krogh, Z. Kutalik, J. M. Lane, Y. Li, Y. Li, P. A. Lind, X. Liu, L. Lu, D. J. MacIntyre, D. F. MacKinnon, R. M. Maier, W. Maier, J. Marchini, H. Mbarek, P. M. Grath, P. M. Guffin, S. E. Medland, D. Mehta, C. M. Middeldorp, E. Mihailov, Y. Milaneschi, L. Milani, J. Mill, F. M. Mondimore, G. W. Montgomery, S. Mostafavi, N. Mullins, M. Nauck, B. Ng, M. G. Nivard, D. R. Nyholt, P. F. O'Reilly, H. Oskarsson, M. J. Owen, J. N. Painter, C. B. Pedersen, M. G. Pedersen, R. E. Peterson, E. Pettersson, W. J. Peyrot, G. Pistis, D. Posthuma, S. M. Purcell, J. A. Quiroz, P. Qvist, J. P. Rice, B. P. Riley, M. Rivera, S. S. Mirza, R. Saxena, R. Schoevers, E. C. Schulte, L. Shen, J. Shi, S. I. Shyn, E. Sigurdsson, G. B. C. Sinnamon, J. H. Smit, D. J. Smith, H. Stefansson, S. Steinberg, C. A. Stockmeier, F. Streit, J. Strohmaier, K. E. Tansey, H. Teismann, A. Teumer, W. Thompson, P. A. Thomson, T. E. Thorgeirsson, C. Tian, M. Traylor, J. Treutlein, V. Trubetskoy, A. G. Uitterlinden, D. Umbricht, S. Van der Auwera, A. M. van Hemert, A. Viktorin, P. M. Visscher, Y. Wang, B. T. Webb, S. M. Weinsheimer, J. Wellmann, G. Willemsen, S. H. Witt, Y. Wu, H. S. Xi, J. Yang, F. Zhang; eQTLGen; 23andMe, V. Arolt, B. T. Baune, K. Berger, D. I. Boomsma, S. Cichon, U. Dannlowski, E. C. J. de Geus, J. R. De Paulo, E. Domenici, K. Domschke, T. Esko, H. J. Grabe, S. P. Hamilton, C. Hayward, A. C. Heath, D. A. Hinds, K. S. Kendler, S. Kloiber, G. Lewis, Q. S. Li, S. Lucae, P. F. A. Madden, P. K. Magnusson, N. G. Martin, A. M. McIntosh, A. Metspalu, O. Mors, P. B. Mortensen, B. Müller-Myhsok, M. Nordentoft, M. M. Nöthen, M. C. O'Donovan, S. A. Paciga, N. L. Pedersen, B. W. J. H. Penninx, R. H. Perlis, D. J. Porteous, J. B. Potash, M. Preisig, M. Rietschel, C. Schaefer, T. G. Schulze, J. W. Smoller, K. Stefansson, H. Tiemeier, R. Uher, H. Völzke, M. M. Weissman, T. Werge, A. R. Winslow, C. M. Lewis, D. F. Levinson, G. Breen, A. D. Børglum, P. F. Sullivan; Major Depressive Disorder Working Group of the Psychiatric Genomics Consortium, Genome-wide association analyses identify 44 risk variants and refine the genetic architecture of major depression. *Nat. Genet.* **50**, 668–681 (2018).

60. S. Benito-Kwiecinski, S. L. Giandomenico, M. Sutcliffe, E. S. Riis, P. Freire-Pritchett, I. Kelava, S. Wunderlich, U. Martin, G. A. Wray, K. McDole, M. A. Lancaster, An early cell shape transition drives evolutionary expansion of the human forebrain. *Cell* **184**, 2084–2102.e19 (2021).

61. M. C. Marchetto, B. Hrvoj-Mihic, B. E. Kerman, D. X. Yu, K. C. Vadodaria, S. B. Linker, I. Narvaiza, R. Santos, A. M. Denli, A. P. Mendes, R. Oefner, J. Cook, L. McHenry, J. M. Grasmick, K. Heard, C. Fredlender, L. Randolph-Moore, R. Kshirsagar, R. Xenitopoulos, G. Chou, N. Hah, A. R. Muotri, K. Padmanabhan, K. Semendeferi, F. H. Gage, Species-specific maturation profiles of human, chimpanzee and bonobo neural cells. *eLife* **8**, e37527 (2019).

62. P. H. Sudmant, T. Rausch, E. J. Gardner, R. E. Handsaker, A. Abyzov, J. Huddleston, Y. Zhang, K. Ye, G. Jun, M. H. Fritz, M. K. Konkel, A. Malhotra, A. M. Stutz, X. Shi, F. P. Casale, J. Chen, F. Hormozdiari, G. Dayama, K. Chen, M. Malig, M. J. P. Chaisson, K. Walter, S. Meiers, S. Kashin, E. Garrison, A. Auton, H. Y. K. Lam, X. J. Mu, C. Alkan, D. Antaki, T. Bae, E. Cerveira, P. Chines, Z. Chong, L. Clarke, E. Dal, L. Ding, S. Emery, X. Fan, M. Gujral, F. Kahveci, J. M. Kidd, Y. Kong, E. W. Lameijer, S. McCarthy, P. Flicek, R. A. Gibbs, G. Marth, C. E. Mason, A. Menelaou, D. M. Muzny, B. J. Nelson, A. Noor, N. F. Parrish, M. Pendleton, A. Quitadamo, B. Raeder, E. E. Schadt, M. Romanovitch, A. Schlattl, R. Sebra, A. A. Shabalin, A. Untergasser, J. A. Walker, M. Wang, F. Yu, C. Zhang, J. Zhang, X. Zheng-Bradley, W. Zhou, T. Zichner, J. Sebat, M. A. Batzer, S. A. McCarroll; 1000 Genomes Project Consortium, R. E. Mills, M. B. Gerstein, A. Bashir, O. Stegle, S. E. Devine, C. Lee, E. E. Eichler, J. O. Korbel, An integrated map of structural variation in 2,504 human genomes. *Nature* **526**, 75–81 (2015).

63. D. A. Grassi, P. L. Brattas, M. E. Jönsson, D. Atacho, O. Karlsson, S. Nolbrant, M. Parmar, J. Jakobsson, Profiling of lincRNAs in human pluripotent stem cell derived forebrain neural progenitor cells. *Heliyon* **6**, e03067 (2020).

64. S. Nolbrant, A. Heuer, M. Parmar, A. Kirkeby, Generation of high-purity human ventral midbrain dopaminergic progenitors for in vitro maturation and intracerebral transplantation. *Nat. Protoc.* **12**, 1962–1979 (2017).

65. R. Zufferey, D. Nagy, R. J. Mandel, L. Naldini, D. Trono, Multiply attenuated lentiviral vector achieves efficient gene delivery in vivo. *Nat. Biotechnol.* **15**, 871–875 (1997).

66. K. J. Livak, T. D. Schmittgen, Analysis of relative gene expression data using real-time quantitative PCR and the $2^{-\Delta\Delta CT}$ method. *Methods* **25**, 402–408 (2001).

67. G. X. Y. Zheng, J. M. Terry, P. Belgrader, P. Ryvkin, Z. W. Bent, R. Wilson, S. B. Ziraldo, T. D. Wheeler, G. P. Mcdermott, J. Zhu, M. T. Gregory, J. Shuga, L. Montesclaros, J. G. Underwood, D. A. Masquelier, S. Y. Nishimura, M. Schnall-Levin, P. W. Wyatt, C. M. Hindson, R. Bharadwaj, A. Wong, K. D. Ness, L. W. Beppu, H. J. Deeg, C. Mcfarland, K. R. Loeb, W. J. Valente, N. G. Ericson, E. A. Stevens, J. P. Radich, T. S. Mikkelsen, B. J. Hindson, J. H. Bielas, Massively parallel digital transcriptional profiling of single cells. *Nat. Commun.* **8**, 14049 (2017).

68. T. Stuart, A. Butler, P. Hoffman, C. Hafemeister, E. Papalexi, W. M. Mauck III, Y. Hao, M. Stoeckius, P. Smibert, R. Satija, Comprehensive integration of single-cell data. *Cell* **177**, 1888–1902.e21 (2019).

69. A. Dobin, C. A. Davis, F. Schlesinger, J. Drenkow, C. Zaleski, S. Jha, P. Batut, M. Chaisson, T. R. Gingeras, STAR: Ultrafast universal RNA-seq aligner. *Bioinformatics* **29**, 15–21 (2013).

70. H. Li, B. Handsaker, A. Wysoker, T. Fennell, J. Ruan, N. Homer, G. Marth, G. Abecasis, R. Durbin; 1000 Genome Project Data Processing Subgroup, The sequence alignment/map format and SAMtools. *Bioinformatics* **25**, 2078–2079 (2009).

71. Y. Liao, G. K. Smyth, W. Shi, featureCounts: An efficient general purpose program for assigning sequence reads to genomic features. *Bioinformatics* **30**, 923–930 (2014).

72. M. I. Love, W. Huber, S. Anders, Moderated estimation of fold change and dispersion for RNA-seq data with *DESeq2*. *Genome Biol.* **15**, 550 (2014).

73. M. Pertea, G. M. Pertea, C. M. Antonescu, T. C. Chang, J. T. Mendell, S. L. Salzberg, StringTie enables improved reconstruction of a transcriptome from RNA-seq reads. *Nat. Biotechnol.* **33**, 290–295 (2015).

74. F. Ramírez, F. Dündar, S. Diehl, B. A. Grüning, T. Manke, DeepTools: A flexible platform for exploring deep-sequencing data. *Nucleic Acids Res.* **42**, W187-W191 (2014).

75. A. Quinlan, I. Hall, BEDTools: A flexible suite of utilities for comparing genomic features. *Bioinformatics* **26**, 841–842 (2010).

76. J. N. Athanikar, R. M. Badge, J. V. Moran, A YY1-binding site is required for accurate human LINE-1 transcription initiation. *Nucleic Acids Res.* **32**, 3846–3855 (2004).

77. H. Li, Minimap2: Pairwise alignment for nucleotide sequences. *Bioinformatics* **34**, 3094–3100 (2018).

78. J. T. Robinson, H. Thorvaldsdóttir, W. Winckler, M. Guttman, E. S. Lander, G. Getz, J. P. Mesirov, Integrative genomics viewer. *Nat. Biotechnol.* **29**, 24–26 (2011).

79. K. L. Spalding, O. Bergmann, K. Alkass, S. Bernard, M. Salehpour, H. B. Huttner, E. Bostrom, I. Westerlund, C. Vial, B. A. Buchholz, G. Possnert, D. C. Mash, H. Druid, J. Frisen, Dynamics of hippocampal neurogenesis in adult humans. *Cell* **153**, 1219–1227 (2013).

80. B. Langmead, S. L. Salzberg, Fast gapped-read alignment with Bowtie 2. *Nat. Methods* **9**, 357–359 (2012).

81. S. Heinz, C. Benner, N. Spann, E. Bertolino, Y. C. Lin, P. Laslo, J. X. Cheng, C. Murre, H. Singh, C. K. Glass, Simple combinations of lineage-determining transcription factors prime cis-regulatory elements required for macrophage and B cell identities. *Mol. Cell* **38**, 576–589 (2010).

82. R. Garza, Y. Sharma, D. Atacho, S. A. Hamdeh, M. Jönsson, M. Ingelsson, P. Jern, M. G. Hammell, E. Englund, J. Jakobsson, N. Marklund, Single-cell transcriptomics of resected human traumatic brain injury tissues reveals acute activation of endogenous retroviruses in oligodendroglia (Cold Spring Harbor Laboratory, 2022).

83. E. K. Gustavsson, S. Sethi, Y. Gao, J. Brenton, S. García-Ruiz, D. Zhang, R. Garza, R. H. Reynolds, J. R. Evans, Z. Chen, M. Grant-Peters, H. Macpherson, K. Montgomery, R. Dore, A. I. Wernick, C. Arber, S. Wray, S. Gandhi, J. Esselborn, C. Blauwendraat, C. H. Douse, A. Adami, D. A. M. Atacho, A. Kouli, A. Quaegebeur, R. A. Barker, E. Englund, F. Platt, J. Jakobsson, N. W. Wood, H. Houlden, H. Saini, C. F. Bento, J. Hardy, M. Ryten, Pseudogenes limit the identification of novel common transcripts generated by their parent genes (Cold Spring Harbor Laboratory, 2022).

84. M. Karimzadeh, C. Ernst, A. Kundaje, M. M. Hoffman, Umap and Bismap: Quantifying genome and methylome mappability. *Nucleic Acids Res.* **46**, e120 (2018).
